# Resolving the fungal velvet domain architecture by *Aspergillus nidulans* VelB

**DOI:** 10.1128/mbio.00261-25

**Published:** 2025-03-31

**Authors:** Wanping Chen, Christoph Sasse, Anna M. Köhler, Dan Wang, Blagovesta Popova, Anja Strohdiek, Gerhard H. Braus

**Affiliations:** 1 Department of Molecular Microbiology and Genetics, Institute of Microbiology and Genetics, Georg-August University, Göttingen, Germany; Nanjing Agricultural University, Nanjing, Jiangsu, China

**Keywords:** *Aspergillus nidulans*, velvet proteins, VeA, VelB, VosA, secondary metabolites, dimerization

## Abstract

Velvet transcription factors are found throughout the fungal kingdom and share a common velvet domain with a fold similar to that of animal NF-κB. They act as homodimeric or heterodimeric master regulators of fungal secondary metabolism, development, and differentiation. A comparison of velvet domains from 4,999 fungal velvet proteins revealed a conserved overall architecture, including an N-terminal DNA-binding region of approximately 30 amino acids and a C-terminal dimerization region of about 100 amino acids. The dimerization region comprises α- and β-subunits separated by a linker region. A detailed analysis of the velvet domain in *Aspergillus nidulans* VelB identified glycine (glycine 240) and leucine (leucine 331) residues in the dimerization region as critical for interactions with velvet proteins. These core amino acid residues are conserved and also essential for the function of VeA or VosA, which corroborates their general importance in functional fungal velvet domains. Functional studies of VelB dimerization linkers suggest its tolerance for length shortening. These findings enhance our understanding of the working mechanisms of fungal velvet regulators.

**IMPORTANCE:**

Fungi, as relatives of animals within Opisthokonta, are closely connected to human life through interactions such as food, pathogenicity, and medicines. Similar to animals, fungi have developed NF-κB-like velvet family regulators to respond to various environmental and internal signals. Velvet regulators form homo- or heterodimers in implementing different functional roles. However, the molecular mechanism by which velvet proteins interact remains incompletely understood. In this study, we found a common architecture of fungal velvet domains and resolved the dimerization region using *Aspergillus nidulans* VelB as a paradigm. The growing understanding of the fungal velvet regulatory network may help to control fungi for pathogenic and industrial purposes and shed light on the general mechanisms shared with the animal NF-κB system in cellular responses to stimuli.

## INTRODUCTION

Fungi play important and distinct roles for human life, as they are essential for the production of food and drugs but can also be harmful, causing serious diseases in humans, plants, or animals (1, 2). Secondary metabolites (SMs) are often implicated in both of these aspects (3). The production of these compounds is closely linked to developmental processes such as sexual and asexual reproductions in fungi, as demonstrated in the ascomycete *Aspergillus nidulans* (4–6). Key regulators for these processes are the velvet proteins (5, 7). This group of regulators is typical for fungi and seems to be restricted to the Opithokonta (8).

Velvet proteins were first discovered in *A. nidulans*, where four different velvet-encoding genes were identified: velvet A (*veA*), velvet-like B (*velB*), velvet-like C (*velC*), and viability of spores A (*vosA*) (5, 9–11). The absence of *veA* leads to the loss of cleistothecia production and altered secondary metabolism (12, 13). A similar phenotype is observed in the absence of *velB* (5, 9), whereas *velC* appears to play a less critical role in developmental processes. The deletion of *velC* causes a slight decrease in sexual fruiting body formation and an increase in the number of conidia (10). VosA is essential for spore viability and acts as a repressor of asexual development (9, 14). Velvet proteins can form both homodimers and various heterodimers with each other, with the VeA-VelB heterodimer being a prominent example. This dimer is shuttled into the nucleus in darkness, where it activates genes required for sexual development (5). In the nucleus, the VelB-VosA dimer is formed, which inhibits asexual development and is required for spore viability (5, 15). In addition to forming different dimers, the velvet complex heterotrimer, consisting of VelB, VeA, and the methyltransferase LaeA, can also be formed in darkness. This heterotrimer coordinates development with secondary metabolism through combined transcriptional and epigenetic control (5). This coordination results in a protective secondary metabolism, including the formation of Hülle cells, which serve as nursing and protecting cells for cleistothecia as sexual fruiting bodies and also function as overwintering structures (5, 16, 17). The multiple interactions of velvet proteins illustrate the complexity of the network regulating development and secondary metabolism, although the complete molecular mechanisms of velvet protein interactions remain incompletely understood.

At least 21 major velvet clades are identified across the fungal kingdom (8). The four-component system of VeA, VelB, VelC, and VosA in aspergilli is the most extensively studied. These four proteins are highly conserved in Pezizomycotina, although their functional roles may differ by species. This study includes a comparison of 4,999 velvet domains across the fungal kingdom. It reveals a common architecture of velvet domains, consisting of an N-terminal DNA-binding region, a variable region, and a C-terminal dimerization region, which includes two subunits separated by a linker. Two conserved amino acid residues essential for VelB dimerization were identified, which are prerequisites for the formation of homo- or heterodimers.

## RESULTS

### The velvet domain is structured in a DNA-binding region, a variable region, and a dimerization region

**Fig 1:**
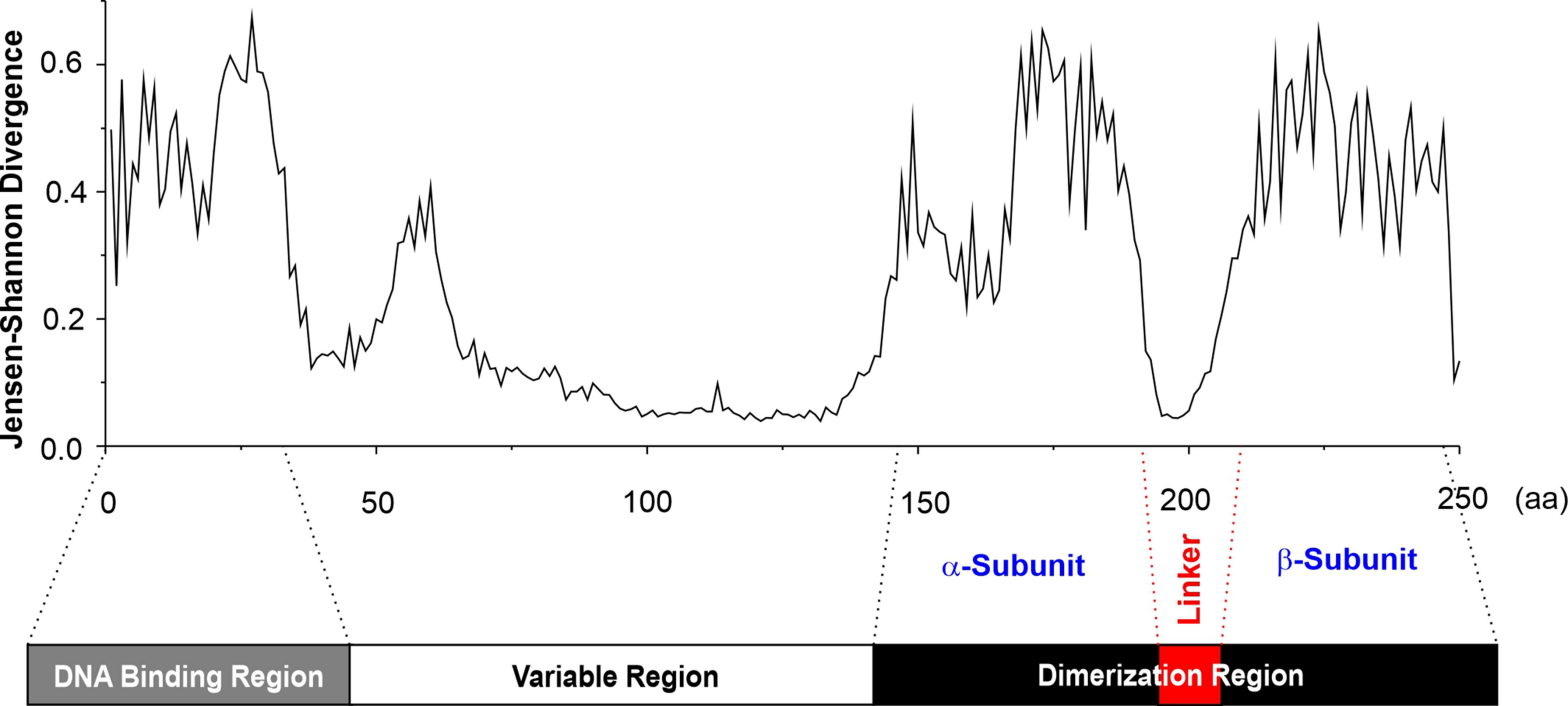
General architecture of fungal velvet domains. Analysis of 4,999 velvet domains across the fungal kingdom revealed a general structure consisting of a DNA-binding region, a variable region, and a dimerization region. The dimerization region is further divided into an α-subunit and a β-subunit, separated by a linker region. The velvet domains of the 4,999 representative velvet proteins across the fungi kingdom (Data S1) were aligned against the hidden Markov model of velvet domain in the Pfam database (http://pfam.xfam.org/family/PF11754). The conservativeness of velvet domains at each position was then calculated using the Jensen–Shannon divergence scoring method, where higher scores indicate greater conservation (19).

The analysis of VosA crystals, combined with functional studies, revealed that velvet domains fold into a highly twisted β-sandwich, with the N-terminal part contributing to DNA binding (18). Conservation at each amino acid position was analyzed *in silico* across 4,999 predicted fungal velvet domains to enhance the understanding of the overall architecture of velvet domains. This analysis indicates that the N-terminal DNA-binding region, approximately 30 amino acids (aa) in length, as well as the C-terminal velvet dimerization region, approximately 100 aa in length, are conserved and connected by a variable region (Fig. 1).

The variable connector region is neither conserved in sequence nor in length and can even be absent. Consequently, the lengths can range from 0 aa to more than 400 aa. The largest group of variable regions, comprising 47% of all analyzed velvet proteins, falls within a range of 40–60 aa (Fig. S1). Large variable regions are typical for basidiomycetes but are uncommon in other fungal taxa. The C-terminal dimerization region contains two conserved parts (referred to as α- and β-subunits), connected by a linker region. The linker length is also variable, with sizes primarily ranging between 15 and 25 aa, found in 63% of the analyzed sequences (Fig. S2). Notably, the architecture of four well-known velvet proteins (VeA, VelB, VelC, and VosA) in *A. nidulans* is illustrated as a reference (Fig. S3). Therefore, the overall conserved structure of velvet domains consists of a DNA-binding region, a variable region, and a dimerization region, which further includes α- and β-subunits separated by a flexible linker.

### Glycine 240 and leucine 331 of the VelB dimerization region are required for accurate development and secondary metabolism

**Fig 2:**
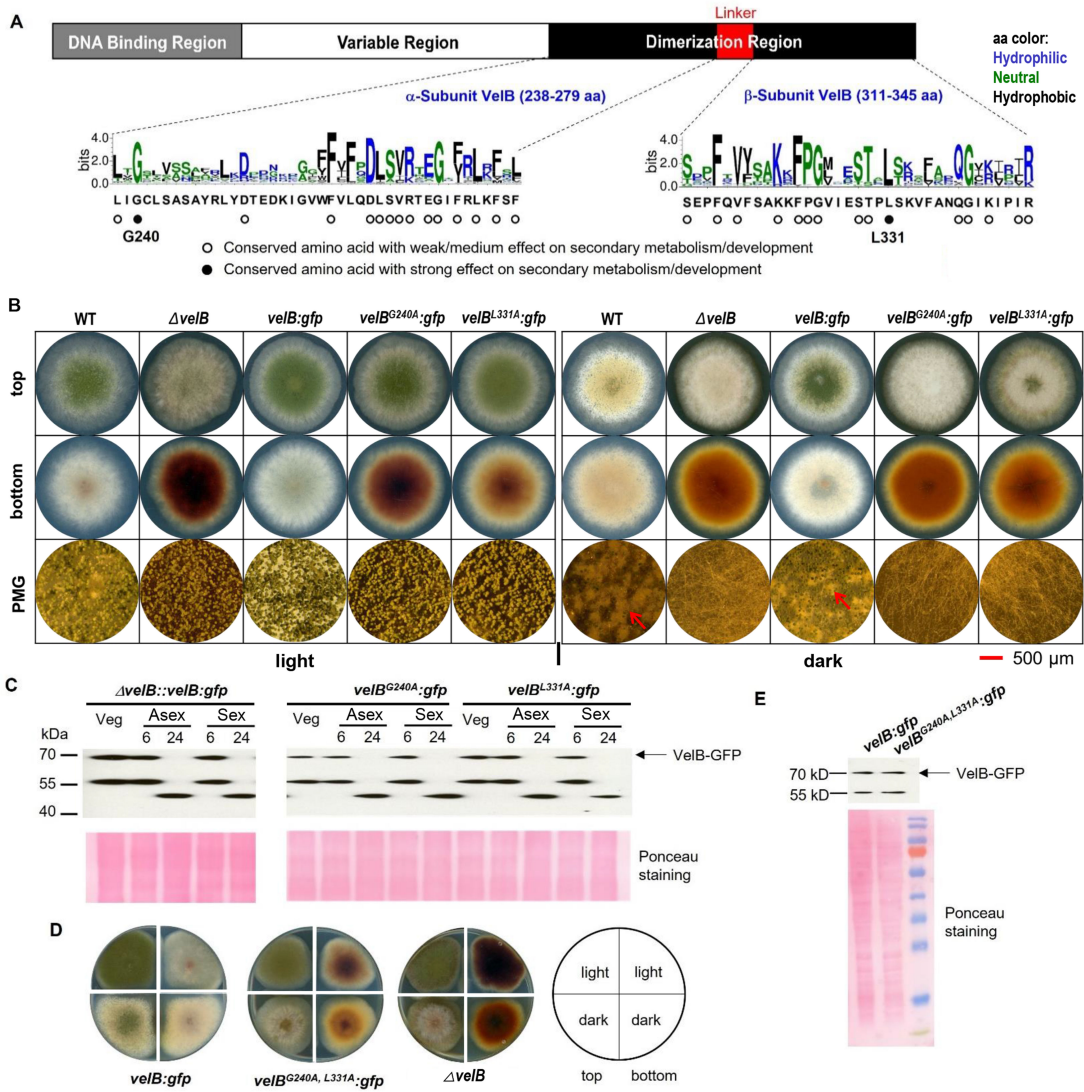
Point mutation of G240A or L331A impairs VelB and leads to defective fungal development in *A. nidulans*. (A) Schematic representation of VelB showing the DNA-binding region, the variable region, and the dimerization region. The dimerization region is subdivided into α-subunit, linker, and β-subunit. Residues are colored by hydrophobicity, with hydrophilic ones in blue, neutral in green, and hydrophobic in black. Conserved aa (indicated by white and black cycles) within the α- and β-subunits were replaced by alanine. (B) Fungal development under asexual (light) and sexual (dark) conditions. The wild type (WT), the *velB* deletion strain, and the VelB-green fluorescent protein (GFP) complementation strain were used as controls. Colony photos were captured on the third day. Red arrows point to sexual fruiting bodies (cleistothecia). The red bar in the lower right is a scale for photomicrograph (PMG). (C) Western blot analysis of VelB^G240A^-GFP and VelB^L331A^-GFP under sexual, asexual, and vegetative growth conditions. The VelB-GFP strain was used as a control. For all conditions, spores were incubated overnight in liquid minimal medium. For sexual and asexual inductions, the mycelium was transferred to solid medium for 6 or 24 h under sexual and asexual conditions, respectively. Protein quantification was performed using Ponceau staining as loading control. (D) Spot test of VelB^G240A, L331A^-GFP under dark and light conditions. The *velB* deletion strain and the VelB-GFP complementation strain were used as controls. (E) Western blot analysis of VelB^G240A, L331A^-GFP under vegetative growth conditions. The VelB-GFP strain was used as a control.

Comparison of the dimerization regions from 4,999 velvet domains identifies 30 conserved aa (threshold: bits over 2) (Fig. 2A). Among the four velvet proteins in *A. nidulans*, VelB has the fewest aa and shows the highest conservation in the fungal kingdom (8), making this protein a paradigm for analyzing the dimerization region. The VelB C-terminal dimerization region consists of an α-subunit and a β-subunit, connected by a linker (Fig. 2A). A general approach to understand the importance of the 30 conserved aa in the α- and β-subunits was to individually replace each corresponding codon with a codon for the small alanine residue in the open reading frame of VelB in *A. nidulans*, given that alanine scanning is a widely recognized technique for identifying critical residues essential for protein function (20).

Several resulting mutant strains exhibited differences from the wild type when their growth phenotypes were compared (Fig. S4; Fig. 2B). Amino acid exchanges primarily influenced colony color during asexual development, suggesting effects on secondary metabolism. The colony color of the *velB^G240A^* mutant closely resembled that of the *velB* deletion strain under asexual conditions. In darkness, which promotes sexual development, the strain with the G240A amino acid exchange displayed the most pronounced phenotype, resembling the *velB* deletion strain and failing to produce sexual fruiting bodies. Several intermediate phenotypes were observed between the wild type and the deletion mutant strain, with L331A being the most notable candidate because it was highly reproducible and the strain among the intermediate phenotype strains with a block in sexual development resulting in hardly any sexual fruiting bodies. Western blot experiments were carried out under different conditions and time points. Both VelB amino acid substitutions showed protein expression patterns similar to the control under vegetative, sexual, and asexual conditions (Fig. 2C). In addition, a mutated *velB^G240A, L331A^* strain carrying both amino acid codon exchanges displayed a phenotype similar to that observed for the strain with the single exchange VelB^G240A^ (Fig. S4; Fig. 2D), and the mutated VelB^G240A, L331A^ protein showed an expression pattern comparable to the control (Fig. 2E).

**Fig 3:**
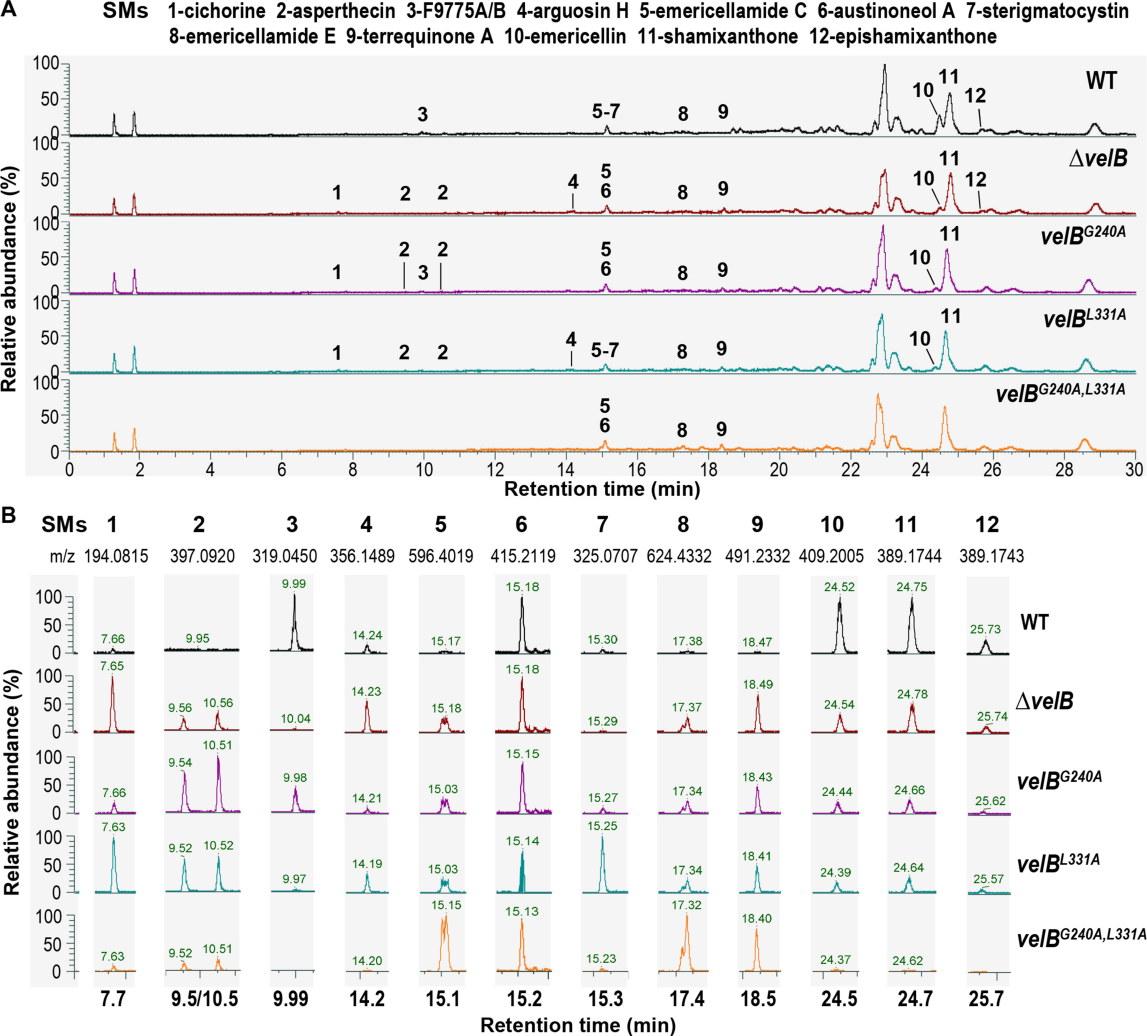
The SM profiles in *A. nidulans* WT and *velB* mutant strains. (A) Charged ion chromatogram of liquid chromatography with tandem mass spectrometry analyzed SM extracts from WT and *velB* mutant strains after 7 days of sexual development. Metabolites were shown as identified when a threshold of 20% relative abundance was reached in the extracted ion chromatogram. The numbers indicate the identified SMs: 1, cichorine (21); 2, asperthecin (22); 3, F9775A/B (23); 4, arguosin H (24); 5, emericellamide C (25); 6, austinoneol A (26); 7, sterigmatocystin (27); 8, emericellamide E (25); 9, terrequinone A (28, 29); 10, emericellin (30); 11, shamixanthone (30); and 12, epishamixanthone (30). (B) Extracted ion chromatograms of 12 SMs. The *m*/*z* of SMs was detected in positive mode. The *m*/*z* of 11 and 12 lacked one water molecule.

The profiles of 12 SMs were compared among the *A. nidulans* wild type and *velB* mutant strains (Fig. 3). The *velB* variants showed an altered secondary metabolism with partially overlapping metabolite profiles to the *velB* deletion strain. Compared to the wild type, the *velB* variants and deletion strain exhibited decreased or no production of emericellin, epishamixanthone, F9775A/B, and shamixanthone while showing increased production of asperthecin, cichorine, emericellamide C, emericellamide E, and terrequinone A. Notably, compared to the wild type, the ∆*velB*, *velB^G240A^*, and *velB^G240A, L331A^* mutants detected no production of sterigmatocystin, whereas the *velB^L331A^* mutant displayed increased production of sterigmatocystin. There was a steady production of austinoneol A among the *A. nidulans* wild type and *velB* mutant strains. These findings indicate that leucine 331 (L331) and glycine 240 (G240) play a prominent role in VelB function to regulate fungal development and secondary metabolism.

### The residues G240 and L331 in the VelB dimerization region are required for homodimerization as well as heterodimer formation with VeA

**Fig 4:**
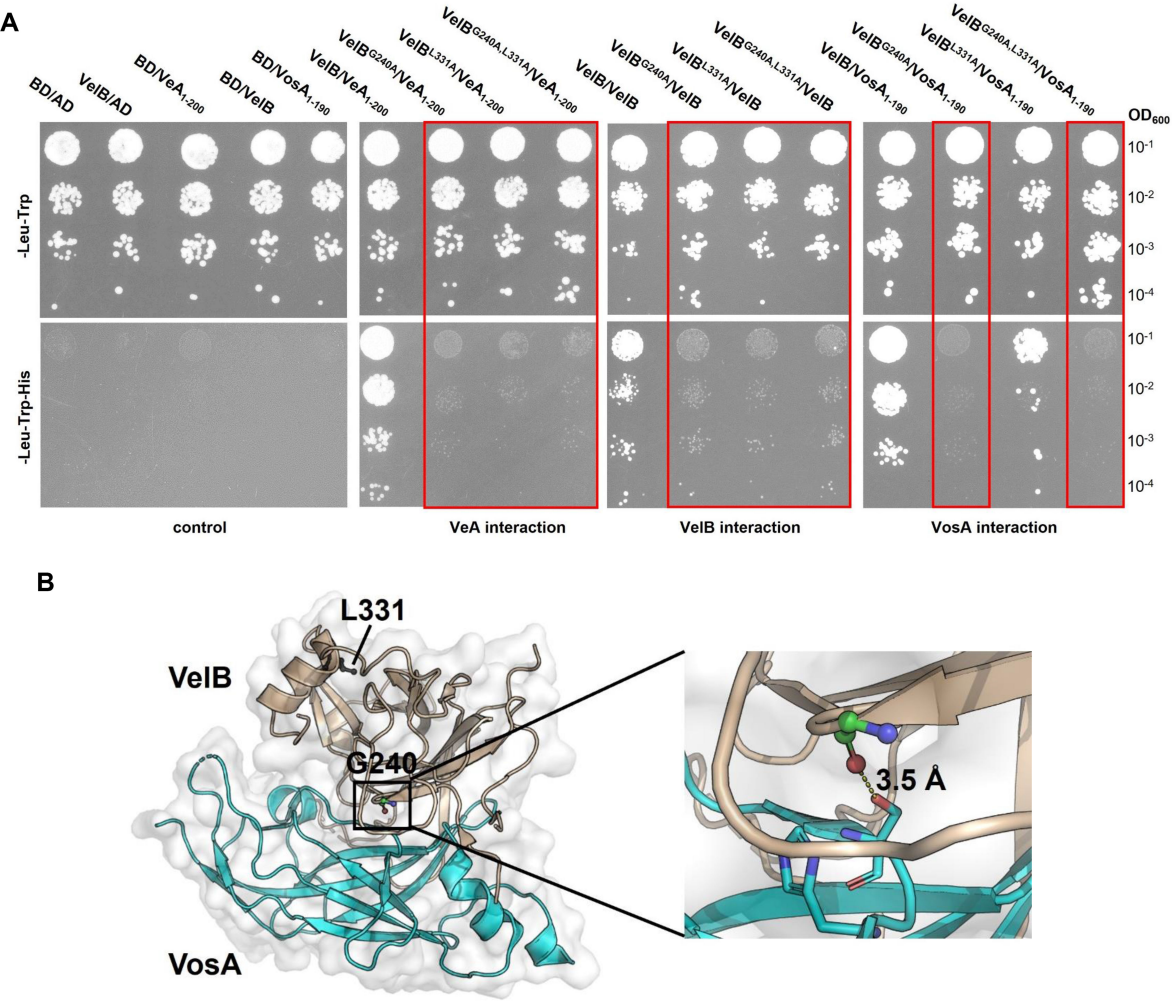
G240 and L331 in VelB are required for interaction with VeA, VelB, and VosA. (A) Yeast two-hybrid assays of VelB variants with VelB, VeA_1–200_, and VosA_1–190_. The genes encoding the velvet proteins were fused with either the activation domain (AD) or the binding domain (BD) of the Gal4 transcription factor, and the derived expression vectors were transformed into the *S. cerevisiae* AH109 strain. Serially diluted yeast cells co-expressing the indicated combinations of constructs were spotted on non-selective (solid synthetic complete dropout [SC]-leucine [Leu]-tryptophan [Trp]) and selective (SC-Leu-Trp-His) media to evaluate the interactions. Upon interaction of the proteins, transcription of the reporter gene *HIS3* is activated, allowing growth on selection medium lacking histidine. Red boxes indicate loss of interaction between proteins with the indicated substitutions. (B) The crystal structure of the VosA-VelB complex (PDB ID: 4N6R) is shown as a cartoon, with VosA and VelB in cyan and wheat, respectively. Two VelB residues, G240 (green) and L331 (black), are depicted as ball-and-stick models. In the enlarged section, G240 (green) forms a hydrogen bond with S110 (cyan, depicted as sticks) of VosA, with a distance of 3.5 Å, indicated by a dashed line.

Amino acid substitutions of G240A and L331A in VelB resulted in altered secondary metabolism and reduced sexual development. Yeast two-hybrid experiments were carried out to investigate whether the G240A and L331A substitutions affect the interaction of VelB with other velvet domain regulators. The interactions of the wild-type VelB protein were compared to those of the VelB variants G240A or L331A, as well as the double substitution G240A and L331A. Shortened versions of VosA and VeA, along with the full-length VelB protein, were tested to focus on the velvet domain as cellular interaction partners for heterodimer or homodimer formation (Fig. 4A). Expression of the different *A. nidulans* VelB variants in the yeast *Saccharomyces cerevisiae* was monitored by Western blot experiments, including the empty bait and prey vectors as controls (Fig. S5). Proteins of the correct size were detected for all constructs. Both VelB single variants (L331A and G240), as well as the double amino acid substitution VelB variant, were impaired in their interactions with VeA as well as with VelB in the yeast two-hybrid experiment, as indicated by growth inhibition on the selection plates (Fig. 4A). In contrast, the interaction with VosA was completely inhibited in the VelB^G240A^-expressing cells but only partially inhibited in the VelB^L331A^-expressing cells (Fig. 4A). While G240 appears to be more critical than L331 for the interaction between VelB and VosA, this is also supported by the crystal structure of the VosA-VelB complex, where the G240 residue directly interacts with VosA by forming a hydrogen bond (Fig. 4B). In conclusion, these data suggest that the amino acid residues G240 and L331 contribute to interactions with velvet proteins. G240 plays an important role in interactions with all three tested velvet proteins, whereas L331 is primarily required for interactions with VeA and VelB, with only a minor effect on VosA interaction. The function of G240 or L331 in dimerization is disrupted by their substitution with alanine.

### The residues G240 and L331 in VeA and VosA are required for full function of velvet proteins

**Fig 5:**
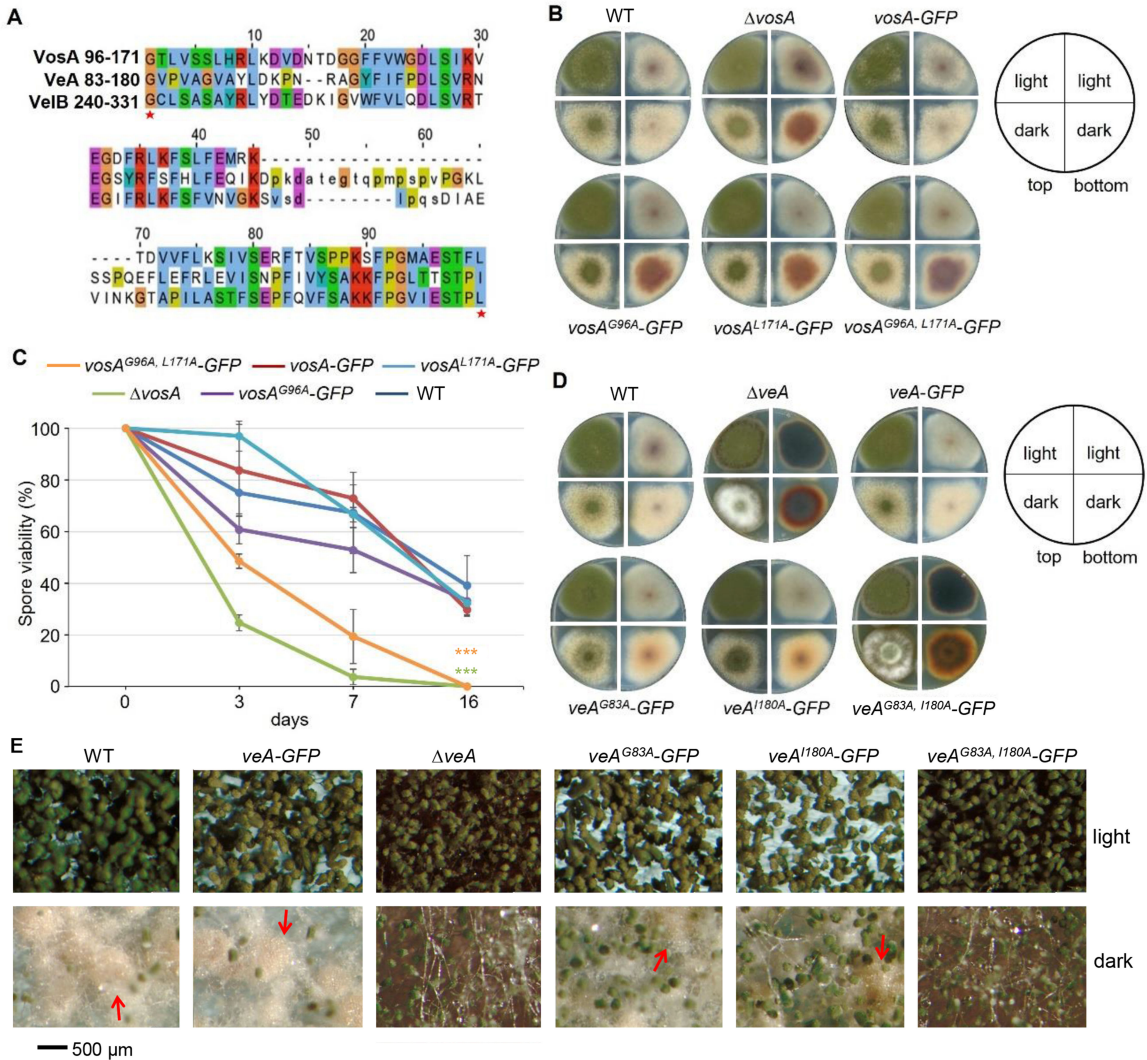
Point mutations in the *A. nidulans vosA* and *veA* dimerization region lead to the corresponding loss of function phenotype. (A) Sequence alignment of *A. nidulans* VeA^83-180^, VelB^240-331^, and VosA^96-171^. Red stars mark the positions of two critical residues for the VelB function. (B) Asexual (light conditions) and sexual (dark conditions) development of the different *vosA* mutant strains. WT, *∆vosA*, and *vosA:gfp* were used for comparison. Colony photos were captured on the fifth day. (C) Spore viability assay. One hundred spores per strain after 0, 3, 7, and 16 days of treatment were plated on minimal medium. The initial spore-forming units were set to 100%. Error bars represent the standard error of the mean from *n* ≥ 3 biological replicates. ***P < 0.0001. The mutant *vosA^G96A,L171A^* showed a rapid loss in long-term spore viability, similar to the *∆vosA*. (D) Asexual and sexual development of the different *veA* mutant strains. WT, *∆veA*, and *veA:gfp* were used for comparison. (E) Photomicrograph on the fifth day for the WT, *∆veA, veA:gfp*, and the *veA* mutated strains. Red arrows point to sexual fruiting bodies. Similar to *∆veA*, no sexual development was observed in the mutant *veA^G83A, I180A^:gfp* under either light or dark conditions.

The residues G240 and L331 in the VelB dimerization region are necessary for interaction with VeA and VelB. Moreover, G240 is also required for interaction with VosA, and to a minor extent, L331 is as well. Alignment of the dimerization region from G240 to L331 of VelB with VosA and VeA reveals that this region is largely conserved across all three proteins. The residues corresponding to VelB G240 and L331 are identified in VosA (at positions G96 and L171). VeA carries a corresponding G83, while at position 180, the leucine is not conserved but replaced by the related amino acid isoleucine (I180) (Fig. 5A). Alanine substitutions were carried out for both corresponding residues in these two proteins to assess the impact of these changes on VeA or VosA function. Both amino acid exchanges, G96A and L171A, as well as the double substitution, result in a *vosA* deletion phenotype, highlighting their importance for the full function of VosA under both sexual and asexual conditions (Fig. 5B). Furthermore, the double substitution mutants lost long-term spore viability, similar to *vosA* knockouts (Fig. 5C). The situation is somewhat different with VeA. Here, individual amino acid exchanges do not lead to strong phenotypes, as observed in the *veA* deletion strain (Fig. 5D). Only the strain with both amino acid exchanges resembles the *veA* deletion strain when cultivated under sexual and asexual development-inducing conditions. In addition to changes in colony color under both tested conditions, the production of sexual structures in dark conditions was blocked as well (Fig. 5E). These results collectively indicate that these two aa are crucial for the function of VeA, VelB, and VosA. Thereby, a single amino acid substitution in VelB and VosA significantly disturbs protein function, whereas in VeA, only the double substitution induces a pronounced phenotypic effect.

### The *A. nidulans* VelB dimerization linker shows a partial tolerance to its length shortening in function

As analyzed (Fig. 1; Fig. S2), the dimerization linker is highly flexible and not conserved in either sequence or length. The dimerization linker of *A. nidulans* VelB consists of 31 residues (positions 280–310) (Fig. S6A). To evaluate the effect of dimerization linker length on velvet function, *A. nidulans velB* mutants with shortened linkers were generated. The growth phenotypes of these *velB* mutants were compared (Fig. S6B). Some mutants exhibited similar or only slightly altered phenotypes compared to the wild type, indicating that the *A. nidulans* VelB dimerization linker can partly tolerate length shortening in function. For an extreme example, the *velB^∆LPQSDIAEVINKGTA289-303^* mutant did not produce any sexual fruiting bodies, similar to the *velB* deletion strain under the tested conditions, while the *velB^∆LPQSDIAEVINKGT289-302^* mutant, with a deletion of 14 linker residues, was still capable of forming sexual fruiting bodies (Fig. S6C), suggesting that the mutated VelB^∆LPQSDIAEVINKGT289-302^ still plays a role in sexual development.

## DISCUSSION

Fungi, as members of the Opisthokonta alongside animals (31), play a significant role in human life through various interactions, including their uses in food production, their capacity as pathogens, and their production of SMs (1–3). Paralleling animal evolutionary strategies, fungi have developed sophisticated NF-κB-like velvet family regulators, which constitute a diverse group of fungal transcription factors characterized by a distinctive velvet domain (8, 18). These velvet regulators can form both homo- and heterodimers, which are essential in orchestrating secondary metabolism, development, and differentiation in fungi (4, 7). Despite their widespread occurrence and functional significance in fungi, the mechanisms by which velvet proteins exert their functions remain largely uncharacterized.

Here we found a general architecture of fungal velvet domains, which includes an N-terminal DNA-binding region, a variable region as well as a C-terminal dimerization region further divided into an α-subunit, a linker, and a β-subunit. The N-terminal DNA-binding region exhibits strong positive potential, as revealed by the electrostatic surface potential of the crystal structures of the VosA homodimer and VosA-VelB heterodimer, and functional studies of this region in *A. nidulans* VosA confirmed the critical roles of four positive residues (K37, K39, R41, and K42) in protein–DNA interactions (18).

Similar to other dimer-forming proteins, such as the Rel/NF-κB transcription factor family (32, 33), the velvet domain contains a conserved dimerization region that facilitates homo- or heterodimerization. This study utilized the *A. nidulans* VelB as a model to resolve the conserved architecture of the velvet domain. Two key residues, G240 and L331, in the dimerization region of VelB were identified through alanine scanning by observing altered phenotypes and secondary metabolism, which are also required for the full function of *A. nidulans* VeA and VosA. It is worth noting that these *velB* mutants obstruct both the development of sexual fruiting bodies (Fig. 2) and the production of the xanthones emericellin, shamixanthone, and epishamixanthone (Fig. 3). These xanthone compounds normally accumulate during sexual development to provide protection for sexual fruiting bodies (16). The roles of G240 and L331 in VelB were further confirmed as critical for homodimerization and heterodimer formation with VeA and VosA. These residues are highly conserved in velvet proteins across the fungal kingdom, with G240 present in 98% and L331 in 95% of the analyzed velvet proteins, suggesting their conserved function. The dimerization region also includes a flexible interdomain linker, which is proposed to adjust dimer pairing. This study reveals a partial tolerance of the *A. nidulans* VelB dimerization linker to its length shortening in function. Therefore, the dimerization region appears to be an important protein interacting platform, where each part plays a specific role in interaction.

In summary, the combination of intensive bioinformatics analysis of 4,999 velvet domains across the fungal kingdom with an initial functional analysis supports the general conserved architecture of velvet domains. Although key residues of velvet domains have been screened by the model of *A. nidulans* VelB, it remains to be clarified how these residue mutants affect the velvet structure and function. Therefore, in the future, more detailed investigations, such as changes in 3D protein structure, nuclear translocation, regulated gene network, and interacting-protein profile of VelB mutants arising from key residue mutations or linker shortening, should be conducted to elaborate on the velvet regulatory network in coordinating fungal development and secondary metabolism as a fungal communication mechanism with the environment.

## MATERIALS AND METHODS

### Search for velvet proteins in the fungal kingdom

Genomic data across the fungal kingdom were accessed from the JGI MycoCosm (https://mycocosm.jgi.doe.gov/mycocosm/home) (34) on 12 May 2021. This included Ascomycota (1,102 genomes), Basidiomycota (567 genomes), Blastocladiomycota (4 genomes), Chytridiomycota (34 genomes), Cryptomycota (2 genomes), Microsporidia (23 genomes), Mucoromycota (101 genomes), and Zoopagomycota (22 genomes). All gene catalog proteins of each genome were used to search for homologs by BlastP (35), with the *A. nidulans* VosA velvet domain (positions 22–186 of VosA protein AN1959) as a query at a threshold lower than E-10. Candidate proteins containing the full velvet domain were selected for further analysis.

### Conservation calculation and sequence logo creation of velvet domains

The filtered 4,999 velvet proteins (Data S1) were aligned by HMMER (version 3.0, http://hmmer.org/) (36) against the velvet domain seed from the Pfam database (PF11754, http://pfam.xfam.org/family/PF11754) (37). Unaligned residues were removed. Sequence alignment conservation was calculated using the scoring method of Jensen–Shannon divergence with default parameters, where higher scores indicate greater conservation (https://compbio.cs.princeton.edu/conservation/score.html) (19). The sequence logo of alignments was generated by WebLogo (version 3, http://weblogo.threeplusone.com/create.cgi) (38). Sequence editing and statistics analyses were performed on Jalview Desktop (http://www.jalview.org/) (39).

### *A. nidulans* and *Escherichia coli* strains and growth conditions

The *A. nidulans* strains used and generated in this study are listed in Table S1. AGB551 (*veA^+^*) was used as the *A. nidulans* wild type. Wild-type and mutant strains were grown in minimal medium (MM) (1% glucose, 7 mM KCl, 2 mM MgSO_4_, 70 mM NaNO_3_, 11.2 mM KH_2_PO_4_, 0.1% trace element solution, pH 5.5) supplemented with 0.1% pyridoxine-HCl, 5 mM uridine, and 5 mM uracil (40, 41). Strains were grown for 3 days on solid MM containing 2% agar in light at 37°C, and 3-day-old spores were harvested for further experiments. For phenotypic observation, around 200 spores were point-inoculated on MM solid plates at 37°C in the dark and sealed, or in light. *E. coli* strains were grown on solid lysogeny broth (LB) medium (1% tryptone, 0.5% yeast extract, and 1% NaCl) or in liquid LB with shaking on a rotary shaker at 37°C (42). Either 100 µg/mL ampicillin or 50 µg/mL kanamycin was added to prevent plasmid loss.

### Oligonucleotides used in this study

The oligonucleotides used in this study are listed in Table S2.

### Genomic DNA extraction

Genomic DNA extraction of *A. nidulans* strains was carried out as previously described (41).

### Plasmid construction and preparation

DNA fragments for plasmid constructions were amplified with PCR and fused into the corresponding enzyme sites of plasmids by usage of the Seamless Cloning and Assembly Kit (Invitrogen). Plasmids were amplified in *E. coli* and extracted with the Qiaprep Spin Miniprep Kit (Qiagen) according to the manufacturer’s instructions. The plasmids constructed and used in this study are listed in Table S3.

### Transformation of *E. coli* and *A. nidulans*

*E. coli* transformations were carried out as described before (43, 44). Successful *E. coli* transformations were confirmed by colony PCR and plasmid sequencing. *A. nidulans* was transformed using polyethylene glycol-mediated protoplast fusion, as previously described (45, 46). The plasmids used for transformation in this study are listed in Table S3, which are with phleomycin or nourseothricin recyclable marker cassettes. The plasmids were first digested by the enzyme *Pme*I, each of which contains two *Pme*I recognition sites. The targeted fragment was recycled, and approximately 5 µg was loaded to incubate with *A. nidulans* protoplasts. Successful transformation of constructs into *A. nidulans* was verified by Southern hybridization. Detailed information on plasmid and mutant generation is provided in the supplemental material.

### Southern blotting

The AlkPhos Direct Labelling and Detection System was employed for Southern hybridization according to the manufacturer’s instructions (GE Healthcare Life Technologies, Little Chalfont, UK).

### Yeast transformation and growth conditions

Yeast transformations for yeast two-hybrid assays are listed in Table S4. Transformation of *S. cerevisiae* with plasmid DNA was performed by the LiAc/SS Carrier DNA/PEG method (47). The strain AH109 (*MATa*, *trp1-901*, *leu2-3*, *112*, *ura3-52*, *his3-200*, *gal4Δ*, *gal80Δ*, *LYS2::GAL1UAS-GAL1TATA-HIS3, GAL2UAS-GAL2TATA-ADE2, URA3::MEL1UAS-MEL1TATA-lacZ*, *MEL1*; Clontech Inc., USA) was grown in non-selective yeast extract-peptone-dextrose medium at 30°C. Co-transformation of two vectors into strain AH109 was performed, and solid synthetic complete dropout (SC) medium lacking amino acids leucine (Leu) and tryptophan (Trp) was used for selection.

### Yeast two-hybrid and spotting assays

Yeast cells used for the two-hybrid assays listed in Table S4 were precultured in liquid SC medium containing 2% raffinose, lacking aa Leu and Trp. After normalizing cell densities to *A*_600_ = 0.1, 10-fold serial dilution series were prepared, and 10 µL of each dilution was spotted onto solid SC-Leu-Trp-His agar plates. Plates were incubated at 30°C to test for interactions.

### Western blot analysis of yeast protein extracts

Western blot analysis of protein extracts from yeast cells expressing VosA_1–190_, VelB, or related mutants in the two-hybrid assay was performed as previously described with modifications (48). Yeast cells were precultured at 30°C in liquid SC-Leu-Trp medium and harvested, and proteins were extracted and denatured using the NaOH lysis/trichloroacetic acid precipitation method (49). Standardized protein samples (20 µL each) were separated on 12% SDS-polyacrylamide gels and transferred to nitrocellulose membranes. Membranes were probed with anti-HA (Sigma-Aldrich, St. Louis, USA) or anti c-Myc mouse antibodies (Santa Cruz Biotechnology, Dallas, USA).

### Protein isolation of *A. nidulans* mycelia

Protein isolation from *A. nidulans* mycelia was performed as previously described (41). Briefly, *A. nidulans* strains were grown under vegetative conditions with 5 × 10^8^ spores in 500 mL liquid MM at 37°C for 20 h. Mycelia were harvested through sterile filter (Merck, Darmstadt, Germany), washed with saline-phenylmethylsulfonyl fluoride (PMSF)-dimethyl sulfoxide, dried, and shock-frozen in liquid nitrogen. Frozen mycelia were ground in liquid nitrogen with a table mill, and B^+^ buffer (300 mM NaCl, 100 mM Tris, pH 7.5, 10% glycerol, 1 mM EDTA, and 0.1% NP-40) supplemented with 1.5 mM dithiothreitol, a complete EDTA-free protease inhibitor cocktail (Roche Diagnostics GmbH, Basel, Switzerland), and 0.001 mM PMSF was added to the grund mycelia in a 1:1 ratio. The mixture was centrifuged for 30 min at 13,000 rpm at 4°C. The supernatant was transferred to fresh test tubes, stored at −20°C, and protein concentration was measured using a NanoDrop ND-1000 spectrophotometer.

### Western blot analysis of *A. nidulans* protein extracts

The GFP fusion proteins were detected as previously reported (41) with some modification. Briefly, the crude protein extract was denatured with 3× loading dye buffer (250 mM Tris-HCl, pH 6.8, 15% [vol/vol] β-mercaptoethanol, 30% [vol/vol] glycerol, 7% [vol/vol] SDS, and 0.3% [wt/vol] bromophenol blue) for each sample. After denaturing the proteins at 95°C for 5 min, approximately 90 µg of denatured crude protein extract from each fungal strain was loaded onto a 12% SDS polyacrylamide gel. Proteins from SDS gels were blotted for 1 h at 100 V onto nitrocellulose membranes (Merck). Ponceau staining was used as loading control, where membranes were incubated in Ponceau S solution (0.2% Ponceau S and 3% acetic acid) for 5 min.

Membranes were subsequently blotted with 5% skim milk powder dissolved in Tris-buffered saline with Tween 20 (TBST) buffer (10 mM Tris-HCl, pH 8.0, 150 mM NaCl, and 0.05% Tween 20) for 1 h at room temperature and then probed with a 1:250 dilution of GFP antibody (sc-9996, Santa Cruz Biotechnology). Following this, membranes were washed three times in TBST, and horseradish peroxidase-coupled mouse antibody (115-035-003, Jackson Immuno Research, West Grove, PA, USA) was applied as the secondary antibody at a dilution of 1:1,000. After incubation for 1 h under agitation at room temperature, the membrane was washed again three times with TBST solution. Solution A (9 mL ddH_2_O, 1 mL 1 M Tris-HCl, pH 8.5, 100 µL 250 mM luminol, and 44 µL 400 mM p-coumaric acid) and solution B (9 mL ddH_2_O, 1 mL 1 M Tris-HCl, pH 8.5, and 6.14 µL 30% H_2_O_2_) were prepared and mixed directly before their addition to the membrane for detection of the chemiluminescence signal. The membrane was gently shaken in solutions A and B at room temperature for 2 min in darkness. The chemiluminescent signals were recorded with the Fusion-SL7 chemiluminescence detection system and analyzed using Fusion software (version 15.18) and Bio1D software (version 15.08).

### Conidiospore quantification

Fresh conidiospores, harvested after 3 days on MM plates, were suspended in a 0.96% NaCl solution containing 0.002% Tween 80, which served as the stock solution. This stock solution could be further diluted to the desired concentration by distilled water. Conidiospore counts were determined using a Coulter Z2 particle counter (Beckman Coulter GmbH, Krefeld, Germany).

### Spore viability assay

The spore viability assay was conducted as previously described, with slight modifications (41). Fresh conidiospores were harvested after 3 days on MM plates and suspended in a 0.96% NaCl solution containing 0.002% Tween 80. The conidiospores were diluted in the same solution to prepare a 10^6^ spores/mL suspension, which was stored at 4℃. To assess viability after treatment at different time points, the stock spore solutions were diluted, and aliquots containing 100 spores from these dilutions were incubated on MM plates for 2 days at 37°C under light conditions. This test was performed in triplicate. The spore viability rate was calculated as (colony count after treatment) × 100/(colony count for fresh spores).

### Extraction of SMs and their liquid chromatography–mass spectrometry analysis

Fresh spores (1 × 10^6^) of each strain were spread on petri dishes containing 30 mL solid MM supplemented with 0.1% pyridoxine-HCl, 5 mM uridine, and 5 mM uracil. The plates were sealed and kept in the dark at 37°C for 7 days. Two agar pieces from the colonies on each plate were cut using a 50 mL Falcon tube and homogenized through a 30 mL syringe. The homogenized agar was mixed with 5 mL of LC-MS grade H_2_O and 5 mL of LC-MS grade ethyl acetate in a 50 mL Falcon tube and shaken at 220 rpm over night at room temperature. Extracts were centrifuged for 5 min at 2,500 rpm. Two milliliters of the upper ethyl acetate phase was transferred into a glass vial and evaporated. The dried extract was stored at −20°C until LC-MS analysis. Three replicates were performed for each strain.

For LC-MS analysis, the extract was dissolved in 500 µL LC-MS-grade acetonitrile:H_2_O (1:1) and centrifuged for 10 min at 13,000 rpm and 4°C. Four hundred microliters of the supernatant was transferred into the LC-MS vial. LC-MS was performed using a Q Exactive Focus Orbitrap mass spectrometer coupled with an UltiMate 3000 high-performance liquid chromatography (HPLC) (Thermo Fisher Scientific, Waltham, USA) equipped with a DAD-3000 Diode Array Detector (Thermo Fisher Scientific) and a Corona Veo RS Charged Aerosol Detector (Thermo Fisher Scientific). A 5 µL sample was injected on an HPLC column (Acclaim 120, C18, 5 µm, 120 Å, 4.6 × 100 mm [Thermo Fisher Scientific]) using a linear acetonitrile/0.1% (vol/vol) formic acid in H_2_O/0.1% (vol/vol) formic acid gradient (from 5% to 95% [vol/vol] acetonitrile/0.1 formic acid in 20 min, followed by an additional 10 min with 95% [vol/vol] acetonitrile/0.1 formic acid) at a flow rate of 0.8 mL/min at 30°C. The measurements were performed in a mass range of 70–1,050 *m*/*z* in both positive and negative modes. Data acquisition and analysis were conducted using Thermo Scientific Xcalibur 4.4 (Thermo Fisher Scientific) and with FreeStyle 1.8SP2 (Thermo Fisher Scientific) software.

### Microscopy

Photomicrographs were captured using an SZX12-ILLB2-200 binocular microscope (Olympus, Tokyo, Japan).

### VosA-VelB structure model based on crystal structure

The crystal structure of the VosA-VelB complex (PDB ID: 4N6R) was generated using the Open-Source version of PyMOL (version 2.6.0a0; copyright Schrödinger, LLC.).

## Data Availability

The data used in the study are provided in the main text or supplemental material.

## SUPPLEMENTAL MATERIAL

Data S1 mbio.00261-25-s0001.txt Sequences of 4,999 velvet proteins.

Supplemental material mbio.00261-25-s0002.pdf Fig. S1 to S6, Tables S1 to S4, and additional experimental details.

ASM does not own the copyrights to Supplemental Material that may be linked to, or accessed through, an article. The authors have granted ASM a non-exclusive, world-wide license to publish the Supplemental Material files. Please contact the corresponding author directly for reuse.
