# Supplementary material for: Resolving the fungal velvet domain architecture by Aspergillus nidulans VelB
Source: mBio. 2025 Mar 31;16(5):e00261-25. doi: 10.1128/mbio.00261-25 (PMC12077163; doi:10.1128/mbio.00261-25)
Supplement: Supplemental material — Fig. S1 to S6, Tables S1 to S4, and additional experimental details. [file mbio.00261-25-s0002.pdf]

Supporting Information for

Resolving the fungal velvet domain architecture by *Aspergillus nidulans* VelB

Wanping Chen[1]*, Christoph Sasse[1,2], Anna M. Köhler[1,3], Dan Wang[1], Blagovesta Popova[1], Anja Strohdiek[1], Gerhard H. Braus[1]*

[1] Department of Molecular Microbiology and Genetics, Institute of Microbiology and Genetics, Georg-August University, Göttingen, Germany

[2] present address: Department of Prosthodontics, University Medical Center, Göttingen, Germany

[3] present address: Institute for Organic Chemistry, Leibniz Universität Hannover, Germany

* Corresponding authors

Wanping Chen (chenwanping1@foxmail.com) and Gerhard H. Braus (gbraus@gwdg.de)

This PDF file includes:

Figures S1 to S6
Tables S1 to S4
Materials and Methods
References

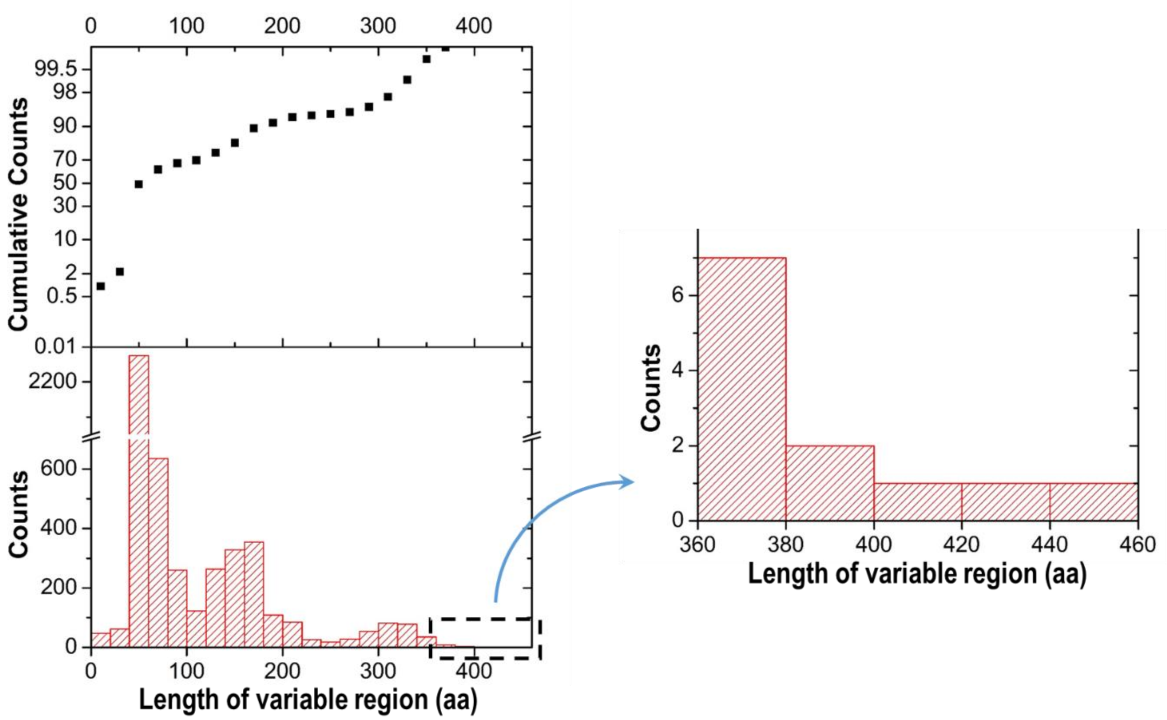

Fig. S1. Length distribution of fungal velvet variable regions. The analysis was based on 4999 deduced velvet proteins (Supplementary data 1). Approximately 47% of fungal velvet domains share a similar lengths of the variable region between 40 and 60 amino acids (aa).

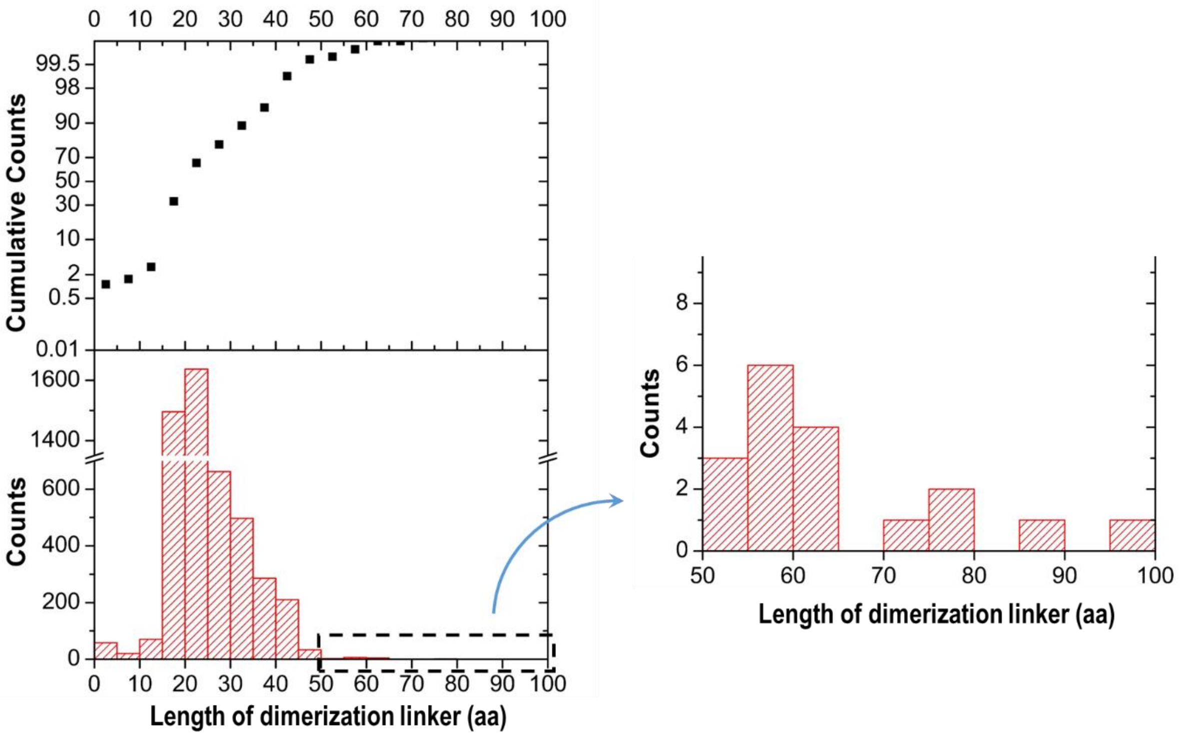

Fig. S2. Length distribution of velvet dimerization linkers. The analysis was based on the 4999 deduced velvet proteins (Supplementary data 1). 63 % of linker lengths are within the range from 15 to 25 aa. The longest linker with 96 aa was detected in the velvet protein (PosplRSB12_1|1049298) of *Phanerochaete chrysosporium*.

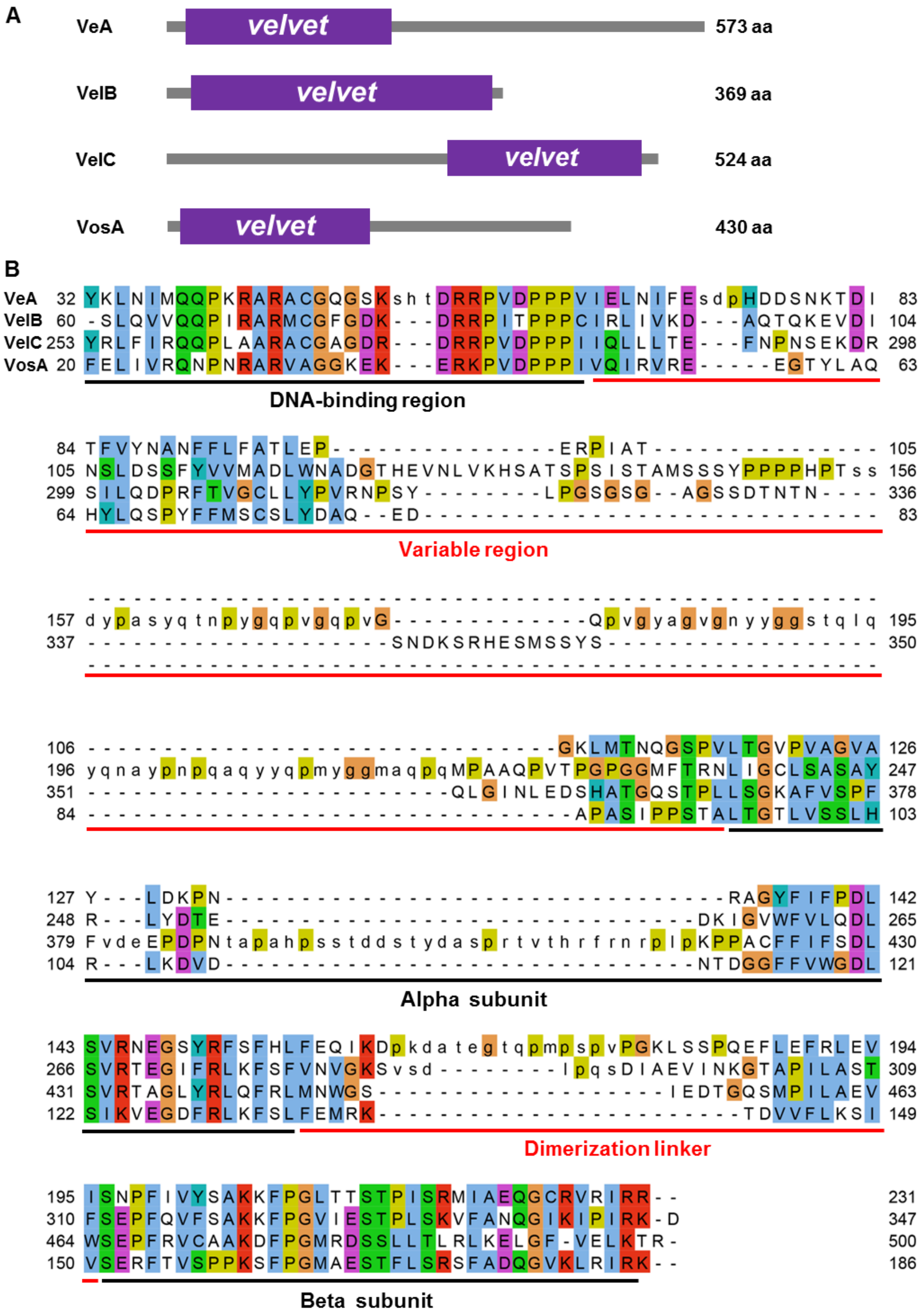

Fig. S3. Structure of the *A. nidulans* VeA, VelB, VelC, and VosA proteins. (A) Position of velvet domain in the proteins. (B) Alignment of the four velvet domains and their functional division.

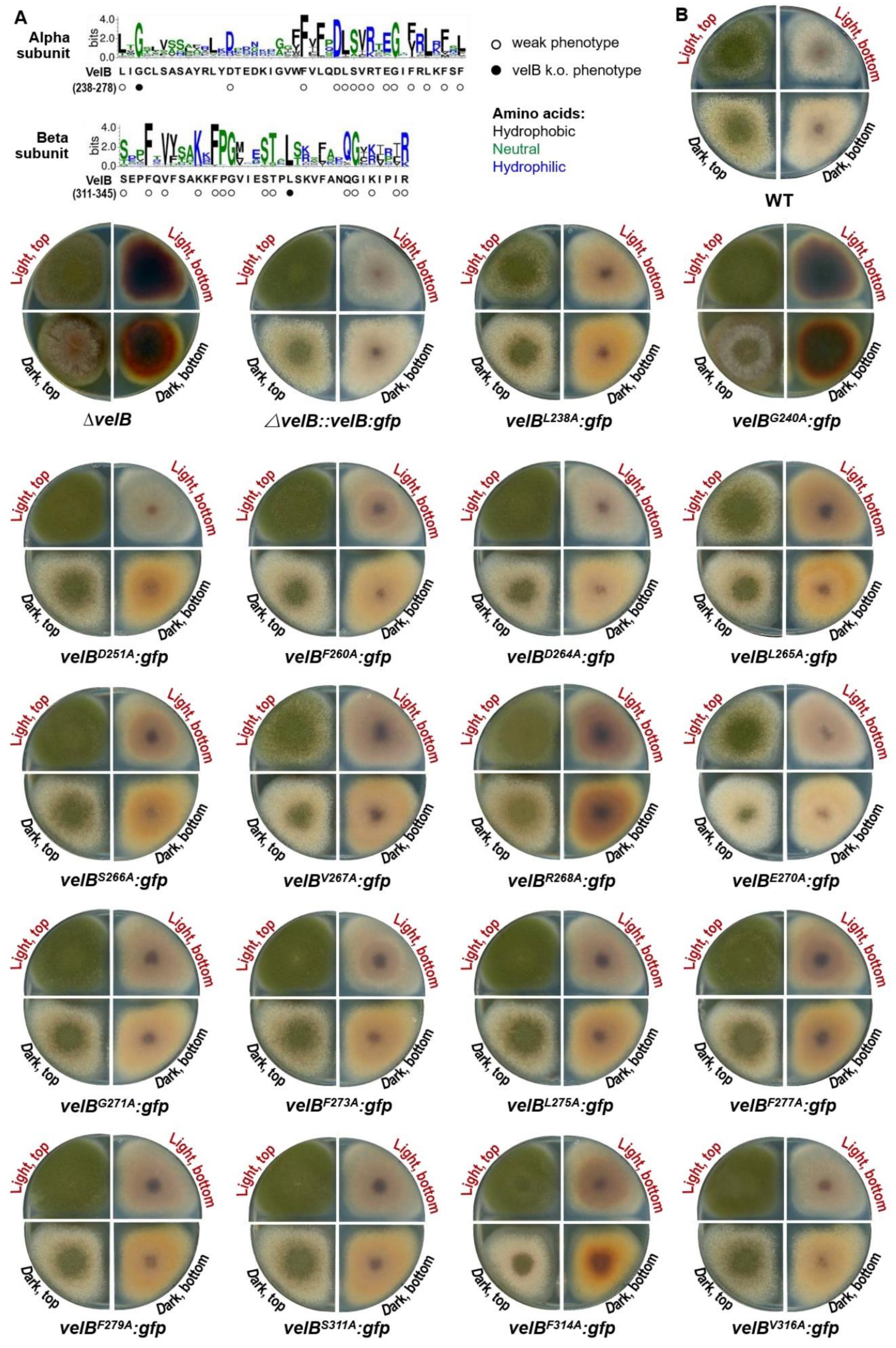

Fig. S4-continued

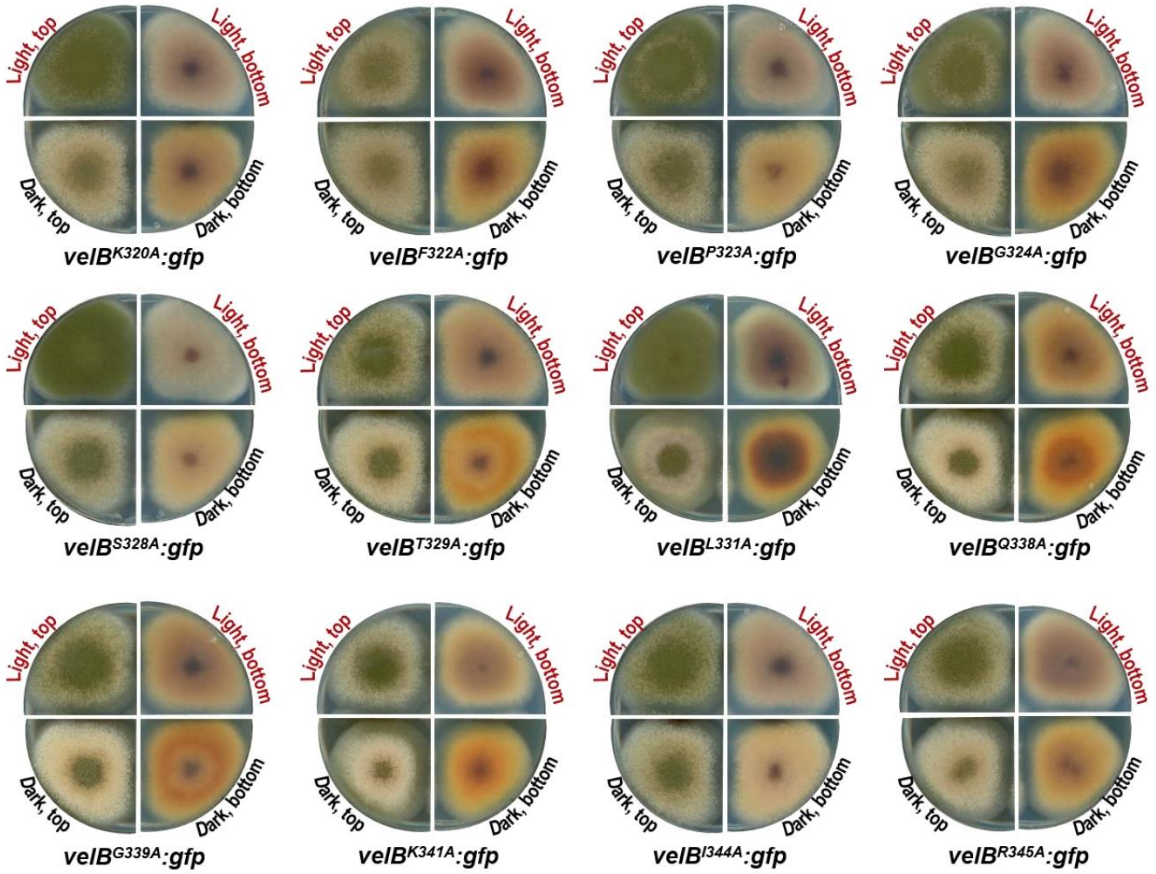

Fig. S4. Alanine codon exchange for different amino acids in the *A. nidulans* VelB dimerization domain. The codons for the indicated amino acids were substituted by alanine codons. (A) Amino acid sequence of the alpha and beta subunit of the dimerization of VelB. The amino acids were classified in neutral, hydrophobic and hydrophilic. (B) The received strains were spotted on minimal medium for 5 days under sexual (dark) and asexual (light) conditions. As control the wildtype, the *velB* deletion strain and *velB-GFP* strain were used.

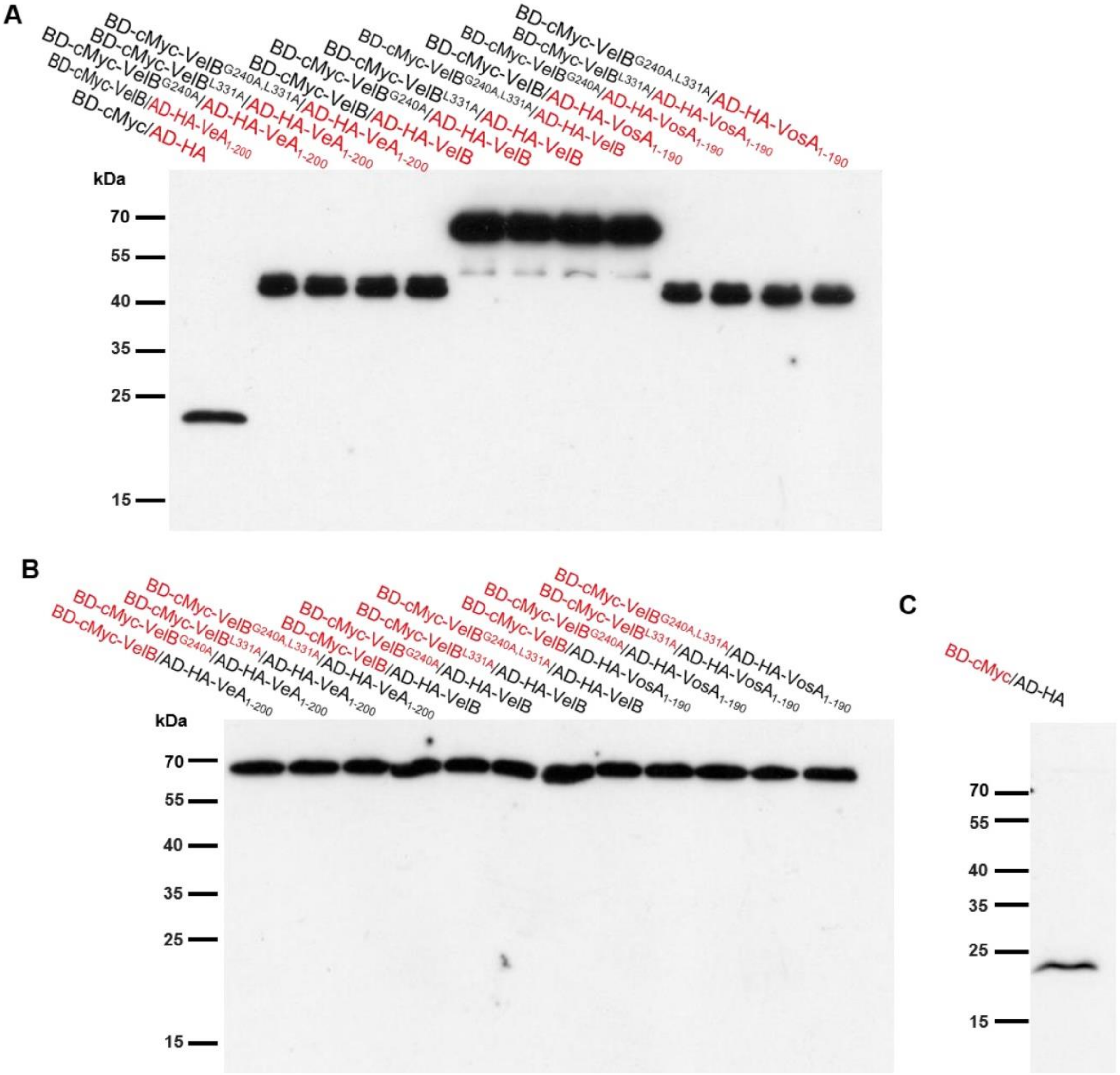

Fig. S5. Western experiments of *A. nidulans* VeA1-200, VosA1-190, VelB and its mutations in yeast two-hybrid assay. (A) Western blot using HA epitope tag antibody. The molecular weights of Gal4 activation domain (AD) with HA tag, AD-HA-VeA1-200, AD-HA-VelB and AD-HA-VosA1-190 are respectively about 21, 43, 61 and 42 kDa. (B and C) Western blot using c-Myc epitope tag antibody. The molecular weights of Gal4 DNA binding domain (BD) with c-Myc tag (C), and BD-cMyc-VelB (B) are respectively about 22, and 62 kDa.

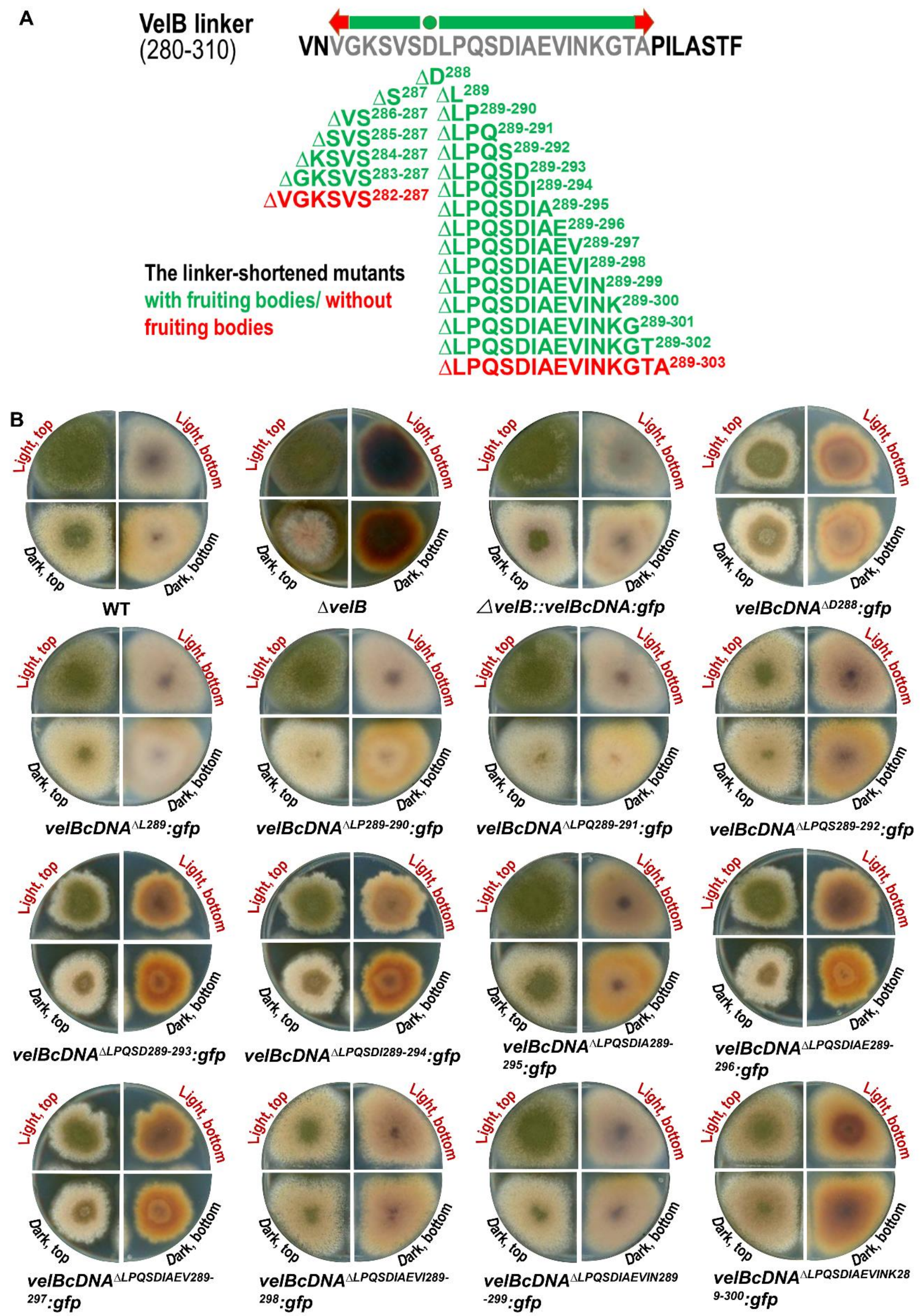

Fig. S6-continued

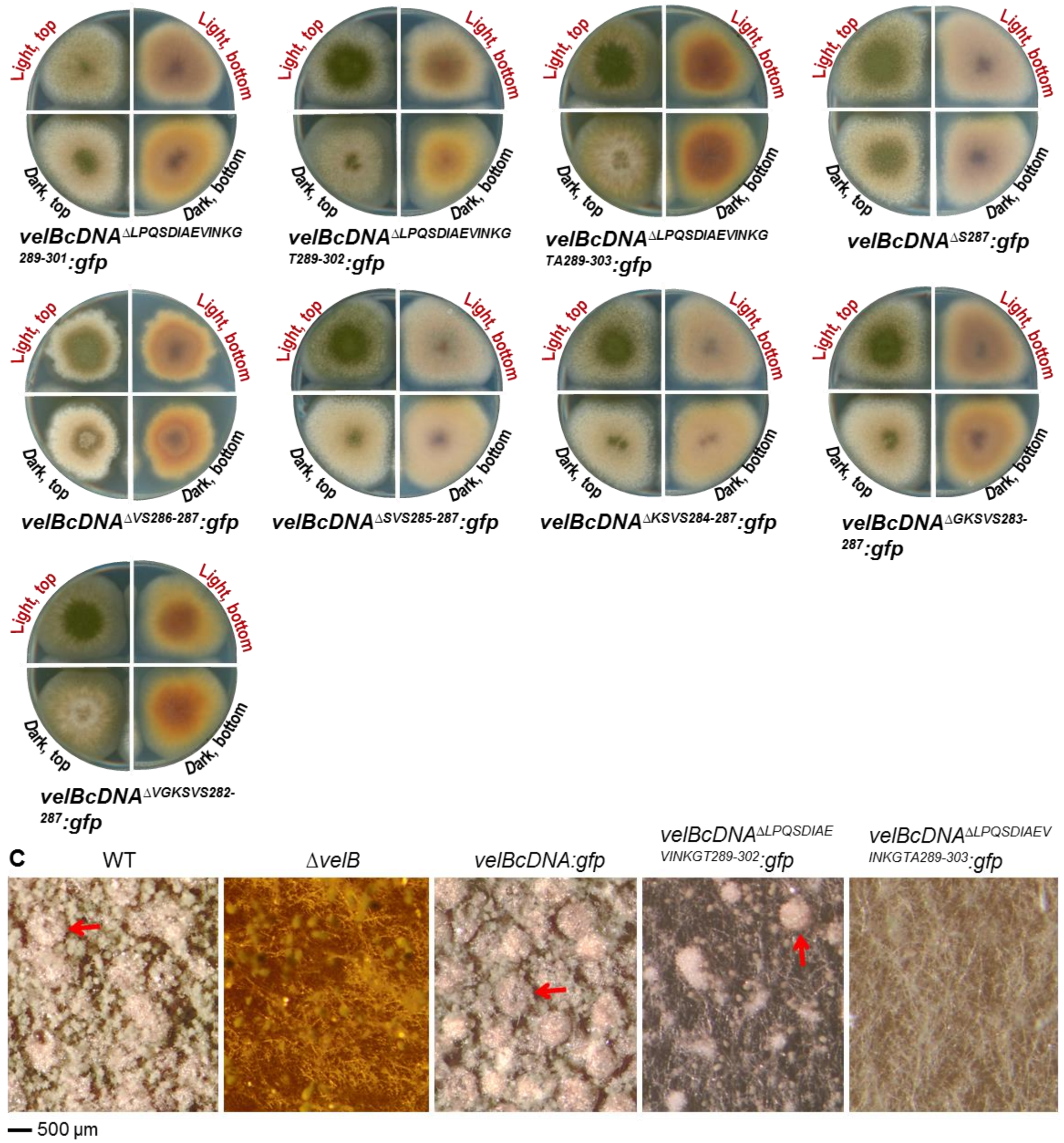

Fig. S6. Shortening of dimerization linker in *A. nidulans* VelB. (A) The impact of dimerization linker shortening on forming of fruiting bodies. (B) The mutanted strains were spotted on minimal medium for 5 days under sexual (dark) and asexual (light) conditions. As control the wildtype, the *velB* deletion strain and velBcDNA-GFP strain were used. (C) Photomicrograph on the fifth day for the WT, ∆*velB*, *velBcDNA:gfp*, *velBcDNA*$^{\Delta LPQSDIAEVINKGT289\text{-}302}$*:gfp*, and *velBcDNA*$^{\Delta LPQSDIAEVINKGTA289\text{-}303}$*:gfp* under dark conditions. Red arrows point to sexual fruiting bodies. Similar to the ∆*velB*, no sexual development was observed in the mutant *velBcDNA*$^{\Delta LPQSDIAEVINKGTA289\text{-}303}$*:gfp* under dark conditions.

Table S1. *A. nidulans* strains used in this study.

Strain name	Genotype	Source or reference
AGB551	*ΔnkuA::argB, pyrG89, pyroA4, veA^{+}*	(1)
AGB1057	*ΔnkuA::argB, pyrG89, pyroA4, veA^{+}, ΔvosA::six*	(2)
AGB1064	*ΔnkuA::argB, pyroA4, pyrG89, veA^{+}, ΔvelB::six*	(2)
AGB1066	*ΔnkuA::argB, pyroA4, pyrG89, veA^{+}, ΔveA::six*	(2)
AGB1165	*ΔnkuA::argB, pyrG89, pyroA4, veA^{+}, veA:sgfp:six*	(3)
AGB1192	*ΔnkuA::argB, pyrG89, pyroA4, veA^{+}, ΔvelB::velB:sgfp:six*	(4)
AGB1194	*ΔnkuA::argB, pyrG89, pyroA4, veA^{+}, ΔvosA::vosA:sgfp:six*	(4)
AGB1479	*ΔnkuA::argB, pyrG89, pyroA4, veA^{+}, ΔvelB::velBcDNA:sgfp:six*	This study
AGB1500	*ΔnkuA::argB, pyrG89, pyroA4, veA^{+}, velB^{L238A}:sgfp:six*	This study
AGB1501	*ΔnkuA::argB, pyrG89, pyroA4, veA^{+}, velB^{G240A}:sgfp:six*	This study
AGB1502	*ΔnkuA::argB, pyrG89, pyroA4, veA^{+}, velB^{D251A}:sgfp:six*	This study

AGB1503	*ΔnkuA::argB, pyrG89, pyroA4, veA*$^{+}$, *velB*^{F260A}*:sgfp:six*	This study
AGB1504	*ΔnkuA::argB, pyrG89, pyroA4, veA*$^{+}$, *velB*^{D264A}*:sgfp:six*	This study
AGB1505	*ΔnkuA::argB, pyrG89, pyroA4, veA*$^{+}$, *velB*^{L265A}*:sgfp:six*	This study
AGB1506	*ΔnkuA::argB, pyrG89, pyroA4, veA*$^{+}$, *velB*^{S266A}*:sgfp:six*	This study
AGB1507	*ΔnkuA::argB, pyrG89, pyroA4, veA*$^{+}$, *velB*^{V267A}*:sgfp:six*	This study
AGB1508	*ΔnkuA::argB, pyrG89, pyroA4, veA*$^{+}$, *velB*^{R268A}*:sgfp:six*	This study
AGB1509	*ΔnkuA::argB, pyrG89, pyroA4, veA*$^{+}$, *velB*^{E270A}*:sgfp:six*	This study
AGB1510	*ΔnkuA::argB, pyrG89, pyroA4, veA*$^{+}$, *velB*^{G271A}*:sgfp:six*	This study
AGB1511	*ΔnkuA::argB, pyrG89, pyroA4, veA*$^{+}$, *velB*^{F273A}*:sgfp:six*	This study
AGB1512	*ΔnkuA::argB, pyrG89, pyroA4, veA*$^{+}$, *velB*^{L275A}*:sgfp:six*	This study
AGB1513	*ΔnkuA::argB, pyrG89, pyroA4, veA*$^{+}$, *velB*^{F277A}*:sgfp:six*	This study
AGB1514	*ΔnkuA::argB, pyrG89, pyroA4, veA*$^{+}$, *velB*^{F279A}*:sgfp:six*	This study

AGB1515	Δ*nkuA::argB, pyrG89, pyroA4, veA*$^{+}$, *velB*^{S311A}*:sgfp:six*	This study
AGB1516	Δ*nkuA::argB, pyrG89, pyroA4, veA*$^{+}$, *velB*^{F314A}*:sgfp:six*	This study
AGB1517	Δ*nkuA::argB, pyrG89, pyroA4, veA*$^{+}$, *velB*^{V316A}*:sgfp:six*	This study
AGB1518	Δ*nkuA::argB, pyrG89, pyroA4, veA*$^{+}$, *velB*^{K320A}*:sgfp:six*	This study
AGB1519	Δ*nkuA::argB, pyrG89, pyroA4, veA*$^{+}$, *velB*^{F322A}*:sgfp:six*	This study
AGB1520	Δ*nkuA::argB, pyrG89, pyroA4, veA*$^{+}$, *velB*^{P323A}*:sgfp:six*	This study
AGB1521	Δ*nkuA::argB, pyrG89, pyroA4, veA*$^{+}$, *velB*^{G324A}*:sgfp:six*	This study
AGB1522	Δ*nkuA::argB, pyrG89, pyroA4, veA*$^{+}$, *velB*^{S328A}*:sgfp:six*	This study
AGB1523	Δ*nkuA::argB, pyrG89, pyroA4, veA*$^{+}$, *velB*^{T329A}*:sgfp:six*	This study
AGB1524	Δ*nkuA::argB, pyrG89, pyroA4, veA*$^{+}$, *velB*^{L331A}*:sgfp:six*	This study
AGB1525	Δ*nkuA::argB, pyrG89, pyroA4, veA*$^{+}$, *velB*^{Q338A}*:sgfp:six*	This study
AGB1526	Δ*nkuA::argB, pyrG89, pyroA4, veA*$^{+}$, *velB*^{G339A}*:sgfp:six*	This study

AGB1527	*ΔnkuA::argB, pyrG89, pyroA4, veA*$^{+}$, *velB*^{K341A}*:sgfp:six*	This study
AGB1528	*ΔnkuA::argB, pyrG89, pyroA4, veA*$^{+}$, *velB*^{I344A}*:sgfp:six*	This study
AGB1529	*ΔnkuA::argB, pyrG89, pyroA4, veA*$^{+}$, *velB*^{R345A}*:sgfp:six*	This study
AGB1540	*ΔnkuA::argB, pyrG89, pyroA4, veA*$^{+}$, *velBcDNA*$^{\Delta D288}$*:sgfp:six*	This study
AGB1541	*ΔnkuA::argB, pyrG89, pyroA4, veA*$^{+}$, *velBcDNA*$^{\Delta L289}$*:sgfp:six*	This study
AGB1542	*ΔnkuA::argB, pyrG89, pyroA4, veA*$^{+}$, *velBcDNA*$^{\Delta LP289\text{-}290}$*:sgfp:six*	This study
AGB1543	*ΔnkuA::argB, pyrG89, pyroA4, veA*$^{+}$, *velBcDNA*$^{\Delta LPQ289\text{-}291}$*:sgfp:six*	This study
AGB1544	*ΔnkuA::argB, pyrG89, pyroA4, veA*$^{+}$, *velBcDNA*$^{\Delta LPQS289\text{-}292}$*:sgfp:six*	This study
AGB1545	*ΔnkuA::argB, pyrG89, pyroA4, veA*$^{+}$, *velBcDNA*$^{\Delta LPQSD289\text{-}293}$*:sgfp:six*	This study
AGB1546	*ΔnkuA::argB, pyrG89, pyroA4, veA*$^{+}$, *velBcDNA*$^{\Delta LPQSDI289\text{-}294}$*:sgfp:six*	This study
AGB1547	*ΔnkuA::argB, pyrG89, pyroA4, veA*$^{+}$, *velBcDNA*$^{\Delta LPQSDIA289\text{-}295}$*:sgfp:six*	This study
AGB1548	*ΔnkuA::argB, pyrG89, pyroA4, veA*$^{+}$, *velBcDNA*$^{\Delta LPQSDIAE289\text{-}296}$*:sgfp:six*	This study

AGB1549	*ΔnkuA::argB, pyrG89, pyroA4, veA⁺, velBcDNA$^{\Delta LPQSDIAEV289-297}$:sgfp:six*	This study
AGB1550	*ΔnkuA::argB, pyrG89, pyroA4, veA⁺, velBcDNA$^{\Delta LPQSDIAEVI289-298}$:sgfp:six*	This study
AGB1551	*ΔnkuA::argB, pyrG89, pyroA4, veA⁺, velBcDNA$^{\Delta LPQSDIAEVIN289-299}$:sgfp:six*	This study
AGB1552	*ΔnkuA::argB, pyrG89, pyroA4, veA⁺, velBcDNA$^{\Delta LPQSDIAEVINK289-300}$:sgfp:six*	This study
AGB1553	*ΔnkuA::argB, pyrG89, pyroA4, veA⁺, velBcDNA$^{\Delta LPQSDIAEVINKG289-301}$:sgfp:six*	This study
AGB1554	*ΔnkuA::argB, pyrG89, pyroA4, veA⁺, velBcDNA$^{\Delta LPQSDIAEVINKGT289-302}$:sgfp:six*	This study
AGB1555	*ΔnkuA::argB, pyrG89, pyroA4, veA⁺, velBcDNA$^{\Delta LPQSDIAEVINKGTA289-303}$:sgfp:six*	This study
AGB1556	*ΔnkuA::argB, pyrG89, pyroA4, veA⁺, velBcDNA$^{\Delta S287}$:sgfp:six*	This study
AGB1557	*ΔnkuA::argB, pyrG89, pyroA4, veA⁺, velBcDNA$^{\Delta VS286-287}$:sgfp:six*	This study
AGB1558	*ΔnkuA::argB, pyrG89, pyroA4, veA⁺, velBcDNA$^{\Delta SVS285-287}$:sgfp:six*	This study
AGB1559	*ΔnkuA::argB, pyrG89, pyroA4, veA⁺, velBcDNA$^{\Delta KSVS284-287}$:sgfp:six*	This study
AGB1560	*ΔnkuA::argB, pyrG89, pyroA4, veA⁺, velBcDNA$^{\Delta GKSVS283-287}$:sgfp:six*	This study

AGB1561	Δ*nkuA::argB, pyrG89, pyroA4, veA*$^{+}$, *velBcDNA*$^{\Delta VGKSVS282\text{-}287}$*:sgfp:six*	This study
AGB1637	Δ*nkuA::argB, pyrG89, pyroA4, veA*$^{+}$, *velB*^{G240A,L331A}*:sgfp:six*	This study
AGB1638	Δ*nkuA::argB, pyrG89, pyroA4, veA*$^{+}$, *veA*^{G83A}*:sgfp:six*	This study
AGB1639	Δ*nkuA::argB, pyrG89, pyroA4, veA*$^{+}$, *veA*^{I180A}*:sgfp:six*	This study
AGB1640	Δ*nkuA::argB, pyrG89, pyroA4, veA*$^{+}$, *veA*^{G83A,I180A}*:sgfp:six*	This study
AGB1641	Δ*nkuA::argB, pyrG89, pyroA4, veA*$^{+}$, *vosA*^{G96A}*:sgfp:six*	This study
AGB1642	Δ*nkuA::argB, pyrG89, pyroA4, veA*$^{+}$, *vosA*^{L171A}*:sgfp:six*	This study
AGB1643	Δ*nkuA::argB, pyrG89, pyroA4, veA*$^{+}$, *vosA*^{G96A,L171A}*:sgfp:six*	This study

Table S2. Oligonucleotides utilized in this study (underlined oligonucleotides were generated the overlaps for seamless cloning in plasmid construction)

Oligo Name	Sequence (5'-3')
WC1	AGGAATTCGATATTTGTTTAAACAGCATCATTTCAGACGCAA
WC5	ATATGGCCATCTCACGGATTCTCGTTTGTGGAACACC
WC6	GATAAGCTTGATCACGTTTAAACTTTCCGTAGGTCGATCCG
WC12	ATAGGCCTGAGATTTCTACTTGTACAGTTCGTCCAT
WC20	ATATGGCCATCTCACAAGAATTCTGCCGGCGTTTATTT
WC21	GATAAGCTTGATCACGTTTAAACATTCTGGCTCGTCTGCATCG
WC22	ATATGGCCATCTCACAGACCGTATATTGTTTCATA
WC23	GATAAGCTTGATCACGTTTAAACCCGCTGTACATGTAATGTCC
WC30	TGCAGGAATTCGATATTTGTTTAAAC
WC31	AAAGCGAAGATACGACCGTCATGCACTGTC
WC54	CGATAAGATTGCGCGTGAACAT
WC55	ACGCGCAATCTTATCGCTTGCTTGAGTGCCAG
WC56	CATACAGCCGGTATGCACTGG
WC57	GCATACCGGCTGTATGCTACAGAGGACAAGAT
WC58	CCATACACCAATCTTGTCCTCTG
WC59	CAAGATTGGTGTATGGGCCGTCTTGCAAGATCT
WC60	CTTGCAAGACGAACCATACACC
WC61	TGGTTCGTCTTGCAAGCTCTTAGTGTGCGAAC

WC62	AAGATCTTGCAAGACGAACCATA
WC63	CGTCTTGCAAGATCTTGCTGTGCGAACAGAAGG
WC64	CACACTAAGATCTTGCAAGACGA
WC65	GCAAGATCTTAGTGTGGCAACAGAAGGAATCTT
WC66	CTTCTGTTCGCACACTAAGATCT
WC67	AGTGTGCGAACAGAAGCAATCTTCCGGTAAGT
WC68	GATTCCTTCTGTTCGCACACTAA
WC69	GCGAACAGAAGGAATCGCCCGGTAAGTTTGACT
WC70	ACTATTTAAGGGTAAGCATGTGG
WC71	CTTACCCTTAAATAGTGCGAAATTCAGTTTTGT
WC72	TTTCAGACTATTTAAGGGTAAGC
WC73	CTTAAATAGTCTGAAAGCCAGTTTTGTCAACGT
WC74	ACTGAATTTCAGACTATTTAAGG
WC75	TAGTCTGAAATTCAGTGCTGTCAACGTCGGCAA
WC76	AAAAGTGCTGGCAAGGATCGG
WC77	CCTTGCCAGCACTTTTGCGGAGCCCTTCCAAG
WC78	GGGCTCCGAAAAAGTGCTGG
WC79	CACTTTTTCGGAGCCCGCCCAAGTCTTTTCAGC
WC80	CTTGGAAGGGCTCCGAAAAAGT
WC81	TCGGAGCCCTTCCAAGCCTTTTCAGCGAAGAA
WC82	CGCTGAAAAGACTTGGAAGGGCT
WC83	CCAAGTCTTTTCAGCGGCGAAGTTCCCTGGTGT

WC84	CTTCTTCGCTGAAAAGACTTGG
WC85	CTTTTCAGCGAAGAAGGCCCCTGGTGTGATTGA
WC86	GAACTTCTTCGCTGAAAAGACTT
WC87	TTCAGCGAAGAAGTTCGCTGGTGTGATTGAAA
WC88	CAGGGAACTTCTTCGCTGAAAA
WC89	GCGAAGAAGTTCCCTGCTGTGATTGAAAGCAC
WC90	TTCAATCACACCAGGGAACTTCT
WC91	CCCTGGTGTGATTGAAGCCACGCCCCTCAGCAA
WC92	GGGCGTGCTTTCAATCACAC
WC93	GATTGAAAGCACGCCCGCCAGCAAAGTCTTTGC
WC94	ATTGCGCGTGAACATGCCTCCA
WC95	CATGTTCACGCGCAATGCTATCGGTTGCTTGAG
WC96	ATCTTGCAAGACGAACCATACAC
WC97	GTTCGTCTTGCAAGATGCTAGTGTGCGAACAGA
WC98	GCTTTCAATCACACCAGGGAAC
WC99	TGGTGTGATTGAAAGCGCGCCCCTCAGCAAAG
WC100	GTTCGCAAAGACTTTGCTGAGG
WC101	CAAAGTCTTTGCGAACGCAGGAATCAAGATCCC
WC102	CTTGGTTCGCAAAGACTTTGCT
WC103	GTCTTTGCGAACCAAGCAATCAAGATCCCCAT
WC104	GATTCCTTGGTTCGCAAAGACT
WC105	TGCGAACCAAGGAATCGCGATCCCCATCCGTAA

WC106	<u>GGGGATCTTGATTCCT</u>TGGTTC
WC107	<u>AGGAATCAAGATCCCC</u>GCCCGTAAGGATGGTGT
WC108	<u>GATGGGGATCTTGATT</u>CCTTGG
WC109	<u>AATCAAGATCCCCATC</u>GCTAAGGATGGTGTCA
WC149	CCAAGTCTTTTCAGCGAAGAAG
WC150	AGGCCCCAGTGCTATGGTT
WC151	AACCATAGCACTGGGGCCT
WC165	GATTGTGGGTCGCTAACGGATTTGCCGACGTT
WC166	TTAGCGACCCACAATCGGATATTGCGGAGGTG
WC167	TCCGATTGGTCGCTAACGGATTTGCCGACGTT
WC168	TTAGCGACCAATCGGATATTGCGGAGGTGATC
WC169	ATATCCGAGTCGCTAACGGATTTGCCGACGTT
WC170	TTAGCGACTCGGATATTGCGGAGGTGATCAAC
WC196	GCAATATCGTCGCTAACGGATTTGCCGACGTT
WC197	TTAGCGACGATATTGCGGAGGTGATCAACAAA
WC198	TCCGCAATGTCGCTAACGGATTTGCCGACGTT
WC199	TTAGCGACATTGCGGAGGTGATCAACAAAGGC
WC200	ACCTCCGCGTCGCTAACGGATTTGCCGACGTT
WC201	TTAGCGACGCGGAGGTGATCAACAAAGGCACT
WC202	TGTGGGAGGCTAACGGATTTGCCGACGTTGAC
WC203	CCGTTAGCCTCCCACAATCGGATATTGCGGAG
WC204	GGGAGGTCAACGGATTTGCCGACGTTGACAAA

WC205	AATCCGTTGACCTCCCACAATCGGATATTGCG
WC206	GGGAGGTCGGATTTGCCGACGTTGACAAAACT
WC207	GCAAATCCGACCTCCCACAATCGGATATTGCG
WC214	ATCACCTCGTCGCTAACGGATTTGCCGACGTT
WC215	TTAGCGACGAGGTGATCAACAAAGGCACTGCG
WC216	TTGATCACGTCGCTAACGGATTTGCCGACGTT
WC217	TTAGCGACGTGATCAACAAAGGCACTGCGCCG
WC218	TTGTTGATGTCGCTAACGGATTTGCCGACGTT
WC219	TTAGCGACATCAACAAAGGCACTGCGCCGATC
WC220	GGGAGGTCTTTGCCGACGTTGACAAAACTGAA
WC221	TCGGCAAAGACCTCCCACAATCGGATATTGCG
WC222	GGGAGGTCGCCGACGTTGACAAAACTGAATTT
WC223	ACGTCGGCGACCTCCCACAATCGGATATTGCG
WC224	GGGAGGTCGACGTTGACAAAACTGAATTTCAG
WC225	TCAACGTCGACCTCCCACAATCGGATATTGCG
WC233	GGGAGGTCGTTGACAAAACTGAATTTCAGACG
WC234	TTGTCAACGACCTCCCACAATCGGATATTGCG
WC239	CCTTTGTTGTCGCTAACGGATTTGCCGACGTT
WC240	TTAGCGACAACAAAGGCACTGCGCCGATCCTT
WC241	GTGCCTTTGTCGCTAACGGATTTGCCGACGTT
WC242	TTAGCGACAAAGGCACTGCGCCGATCCTTGCC
WC243	GCAGTGCCGTCGCTAACGGATTTGCCGACGTT

WC244	TTAGCGACGGCACTGCGCCGATCCTTGCCAGC
WC245	GGCGCAGTGTCGCTAACGGATTTGCCGACGTT
WC246	TTAGCGACACTGCGCCGATCCTTGCCAGCACT
WC247	ATCGGCGCGTCGCTAACGGATTTGCCGACGTT
WC248	TTAGCGACGCGCCGATCCTTGCCAGCACTTTT
WC249	AGGATCGGGTCGCTAACGGATTTGCCGACGTT
WC250	TTAGCGACCCGATCCTTGCCAGCACTTTTTCG
WC253	ACTAAGATCTTGCAAGACGAACCATAC
WC254	TTGCAAGATCTTAGTGCGCGAACAGAAGGAAT
WC255	TGTTCGCACACTAAGATCTTGCAAG
WC256	CTTAGTGTGCGAACAGCAGGAATCTTCCGGTA
WC278	TGGAGGCCGAATTCCCGTACGCTGTTGAGGATAGGGCG
WC279	AGGTCGACGGATCCCCGTATTCGTTATCCAGACCATCG
WC282	CCAGTGAATTCCACCCGTACGCTGTTGAGGATAGGGCG
WC283	GTATCGATGCCCACCCGTATTCGTTATCCAGACCATCG
WC284	CCAGTGAATTCCACCCGAGTGCGGCGAACTATCCAGAT
WC285	GTATCGATGCCCACCCTGTGCGCGGCTCCTTTCGAATC
WC286	CCAGTGAATTCCACCCGCAGCAGCCCAAGCGCGCGAGAGC
WC287	GTATCGATGCCCACCCTCTCATCCGCACATCGCGCCGGAT
WC289	CTGTCAGGACCGGCGAGCCTTGGT
WC290	TCGCCGGTCCTGACAGCCGTCCCGGTAGCTGG
WC291	GGGGGTGCTCGTGGTCAGCCCAGG

WC292	GACCACGAGCACCCCCGCCAGCCGTATGATTGC
WC293	CAGTCAATGCAGTAGACGGTGGAA
WC294	TCTACTGCATTGACTGCGACTCTGGTTTCGTC
WC295	GAAGGTTGATTCGGCCATCCCCGG
WC296	GGCCGAATCAACCTTCGCTTCCAGATCCTTCGC

Table S3. Plasmids constructed and used in this study. natRM = nourseothricin recyclable marker cassette, phleoRM = phleomycin recyclable marker cassette

Plasmid	Description	Source
pGADT7	*2μ; LEU2; ADH1pr; ADH1term; AmpR*	Clontech Laboratories, Inc.
pGBKT7	*2μ; TRP1; ADH1pr; ADH1term; KanR*	Clontech Laboratories, Inc.
pME3189	^{p}*niiA::n-eyfp::veA /* ^{p}*niiD::c-eyfp::velB, pyrG*	(5)
pME3715	^{p}*niiA*::*c-yfp::velB*/^{p}*niiD::n-yfp::vosA*, *pyrG*	(6)
pME3717	^{p}*niiA::n-yfp::velB/pniiD::c-yfp::velB, pyrG* (BIFC 3)	(6)
pME4319	The recyclable marker cassette-containing plasmid with the bleo gene conferring resistance to phleomycin	(7)
pME4687	*A. nidulans velB* with *sgfp tag: velB::sgfp::natRM*	(4)
pME4714	*A. nidulans veA* with *sgfp tag: veA:sgfp::natRM*	(3)
pME4749	*A. nidulans vosA* with *sgfp tag: vosA:sgfp::natRM*	(4)
pME5332	*A. nidulans velB* 3UTR integrated in the PmlI restriction cutting site of plasmid pME4319	This study

pME5333	*A. nidulans velB_5UTR:velBcDNA: sgfp: phleoRM:velB_3UTR*	This study
pME5354	*A. nidulans velB_5UTR: velB^{L238A}:sgfp: phleoRM: velB_3UTR*	This study
pME5355	*A. nidulans velB_5UTR: velB^{G240A}:sgfp: phleoRM: velB_3UTR*	This study
pME5356	*A. nidulans velB_5UTR: velB^{D251A}:sgfp: phleoRM: velB_3UTR*	This study
pME5357	*A. nidulans velB_5UTR: velB^{F260A}:sgfp: phleoRM: velB_3UTR*	This study
pME5358	*A. nidulans velB_5UTR: velB^{D264A}:sgfp: phleoRM: velB_3UTR*	This study
pME5359	*A. nidulans velB_5UTR: velB^{L265A}:sgfp: phleoRM: velB_3UTR*	This study
pME5360	*A. nidulans velB_5UTR: velB^{S266A}:sgfp: phleoRM: velB_3UTR*	This study
pME5361	*A. nidulans velB_5UTR: velB^{V267A}:sgfp: phleoRM: velB_3UTR*	This study
pME5362	*A. nidulans velB_5UTR: velB^{R268A}:sgfp: phleoRM: velB_3UTR*	This study
pME5363	*A. nidulans velB_5UTR: velB^{E270A}:sgfp: phleoRM: velB_3UTR*	This study
pME5364	*A. nidulans velB_5UTR: velB^{G271A}:sgfp: phleoRM: velB_3UTR*	This study

pME5365	*A. nidulans velB_5UTR: velB^{F273A}:sgfp: phleoRM: velB_3UTR*	This study
pME5366	*A. nidulans velB_5UTR: velB^{L275A}:sgfp: phleoRM: velB_3UTR*	This study
pME5367	*A. nidulans velB_5UTR: velB^{F277A}:sgfp: phleoRM: velB_3UTR*	This study
pME5368	*A. nidulans velB_5UTR: velB^{F279A}:sgfp: phleoRM: velB_3UTR*	This study
pME5369	*A. nidulans velB_5UTR: velB^{S311A}:sgfp: phleoRM: velB_3UTR*	This study
pME5370	*A. nidulans velB_5UTR: velB^{F314A}:sgfp: phleoRM: velB_3UTR*	This study
pME5371	*A. nidulans velB_5UTR: velB^{V316A}:sgfp: phleoRM: velB_3UTR*	This study
pME5372	*A. nidulans velB_5UTR: velB^{K320A}:sgfp: phleoRM: velB_3UTR*	This study
pME5373	*A. nidulans velB_5UTR: velB^{F322A}:sgfp: phleoRM: velB_3UTR*	This study
pME5374	*A. nidulans velB_5UTR: velB^{P323A}:sgfp: phleoRM: velB_3UTR*	This study
pME5375	*A. nidulans velB_5UTR: velB^{G324A}:sgfp: phleoRM: velB_3UTR*	This study
pME5376	*A. nidulans velB_5UTR: velB^{S328A}:sgfp: phleoRM: velB_3UTR*	This study

pME5377	*A. nidulans velB_5UTR: velB^{T329A}:sgfp: phleoRM: velB_3UTR*	This study
pME5378	*A. nidulans velB_5UTR: velB^{L331A}:sgfp: phleoRM: velB_3UTR*	This study
pME5379	*A. nidulans velB_5UTR: velB^{Q338A}:sgfp: phleoRM: velB_3UTR*	This study
pME5380	*A. nidulans velB_5UTR: velB^{G339A}:sgfp: phleoRM: velB_3UTR*	This study
pME5381	*A. nidulans velB_5UTR: velB^{K341A}:sgfp: phleoRM: velB_3UTR*	This study
pME5382	*A. nidulans velB_5UTR: velB^{I344A}:sgfp: phleoRM: velB_3UTR*	This study
pME5383	*A. nidulans velB_5UTR: velB^{R345A}:sgfp: phleoRM: velB_3UTR*	This study
pME5393	*A. nidulans velB_5UTR: velBcDNA$^{\Delta D288}$:sgfp: phleoRM: velB_3UTR*	This study
pME5394	*A. nidulans velB_5UTR: velBcDNA$^{\Delta L289}$:sgfp: phleoRM: velB_3UTR*	This study
pME5395	*A. nidulans velB_5UTR: velBcDNA$^{\Delta LP289-290}$:sgfp: phleoRM: velB_3UTR*	This study
pME5396	*A. nidulans velB_5UTR: velBcDNA$^{\Delta LPQ289-291}$:sgfp: phleoRM: velB_3UTR*	This study
pME5397	*A. nidulans velB_5UTR: velBcDNA$^{\Delta LPQS289-292}$:sgfp: phleoRM: velB_3UTR*	This study

pME5398	*A. nidulans velB_5UTR: velBcDNA*$^{\Delta LPQSD289\text{-}293}$*:sgfp: phleoRM: velB_3UTR*	This study
pME5399	*A. nidulans velB_5UTR: velBcDNA*$^{\Delta LPQSDI289\text{-}294}$*:sgfp: phleoRM: velB_3UTR*	This study
pME5400	*A. nidulans velB_5UTR: velBcDNA*$^{\Delta LPQSDIA289\text{-}295}$*:sgfp: phleoRM: velB_3UTR*	This study
pME5401	*A. nidulans velB_5UTR: velBcDNA*$^{\Delta LPQSDIAE289\text{-}296}$*:sgfp: phleoRM: velB_3UTR*	This study
pME5402	*A. nidulans velB_5UTR: velBcDNA*$^{\Delta LPQSDIAEV289\text{-}297}$*:sgfp: phleoRM: velB_3UTR*	This study
pME5403	*A. nidulans velB_5UTR: velBcDNA*$^{\Delta LPQSDIAEVI289\text{-}298}$*:sgfp: phleoRM: velB_3UTR*	This study
pME5404	*A. nidulans velB_5UTR: velBcDNA*$^{\Delta LPQSDIAEVIN289\text{-}299}$*:sgfp: phleoRM: velB_3UTR*	This study
pME5405	*A. nidulans velB_5UTR: velBcDNA*$^{\Delta LPQSDIAEVINK289\text{-}300}$*:sgfp: phleoRM: velB_3UTR*	This study
pME5406	*A. nidulans velB_5UTR: velBcDNA*$^{\Delta LPQSDIAEVINKG289\text{-}301}$*:sgfp: phleoRM: velB_3UTR*	This study
pME5407	*A. nidulans velB_5UTR: velBcDNA*$^{\Delta LPQSDIAEVINKGT289\text{-}302}$*:sgfp: phleoRM: velB_3UTR*	This study

pME5408	*A. nidulans velB_5UTR: velBcDNA*$^{\Delta LPQSDIAEVINKGTA289\text{-}303}$*:sgfp: phleoRM: velB_3UTR*	This study
pME5409	*A. nidulans velB_5UTR: velBcDNA*$^{\Delta S287}$*:sgfp: phleoRM: velB_3UTR*	This study
pME5410	*A. nidulans velB_5UTR: velBcDNA*$^{\Delta VS286\text{-}287}$*:sgfp: phleoRM: velB_3UTR*	This study
pME5411	*A. nidulans velB_5UTR: velBcDNA*$^{\Delta SVS285\text{-}287}$*:sgfp: phleoRM: velB_3UTR*	This study
pME5412	*A. nidulans velB_5UTR: velBcDNA*$^{\Delta KSVS284\text{-}287}$*:sgfp: phleoRM: velB_3UTR*	This study
pME5413	*A. nidulans velB_5UTR: velBcDNA*$^{\Delta GKSVS283\text{-}287}$*:sgfp: phleoRM: velB_3UTR*	This study
pME5414	*A. nidulans velB_5UTR: velBcDNA*$^{\Delta VGKSVS282\text{-}287}$*:sgfp: phleoRM: velB_3UTR*	This study
pME5464	*pGADT7-GAL4-AD-VeA*$_{1\text{-}200}$	This study
pME5465	*pGADT7-GAL4-AD-VelB*	This study
pME5466	*pGADT7-GAL4-AD-VosA*$_{1\text{-}190}$	This study
pME5467	*pGBKT7-GAL4-BD-VelB*	This study
pME5468	*pGBKT7-GAL4-BD-VelB*^{G240A}	This study
pME5469	*pGBKT7-GAL4-BD-VelB*^{L331A}	This study
pME5470	*pGBKT7-GAL4-BD-VelB*^{G240A,L331A}	This study
pME5471	*A. nidulans velB_5UTR: velB*^{G240A,L331A}*:sgfp: phleoRM: velB_3UTR*	This study

pME5472	*A. nidulans veA_5UTR: veA^{G83A}:sgfp: phleoRM: veA_3UTR*	This study
pME5473	*A. nidulans veA_5UTR: veA^{I180A}:sgfp: phleoRM: veA_3UTR*	This study
pME5474	*A. nidulans veA_5UTR: veA^{G83A,I180A}:sgfp: phleoRM: veA_3UTR*	This study
pME5475	*A. nidulans vosA_5UTR: vosA^{G96A}:sgfp: phleoRM: vosA_3UTR*	This study
pME5476	*A. nidulans vosA_5UTR: vosA^{L171A}:sgfp: phleoRM: vosA_3UTR*	This study
pME5477	*A. nidulans vosA_5UTR: vosA^{G96A,L171A}:sgfp: phleoRM: vosA_3UTR*	This study

Table S4. Yeast transformations for yeast two-hybrid assays.

Name	Genotype	Source
AH109-BD-AD	AH109 harbors vectors pGADT7 (GAL4-AD) and pGBKT7 (GAL4-BD).	This study
AH109-VelB-AD	AH109 harbors vectors pGADT7 (GAL4-AD) and pME5467 (GAL4-BD-VelB).	This study
AH109-BD-VeA$_{1-200}$	AH109 harbors vectors pME5464 (GAL4-AD-VeA$_{1-200}$) and pGBKT7 (GAL4-BD).	This study
AH109-BD-VelB	AH109 harbors vectors pME5465 (GAL4-AD-VelB) and pGBKT7 (GAL4-BD).	This study
AH109-BD-VosA$_{1-190}$	AH109 harbors vectors pME5466 (GAL4-AD-VosA$_{1-190}$) and pGBKT7 (GAL4-BD).	This study
AH109-VelB-VeA$_{1-200}$	AH109 harbors vectors pME5464 (GAL4-AD-VeA$_{1-200}$) and pME5467 (GAL4-BD-VelB).	This study
AH109-VelB^{G240A}-VeA$_{1-200}$	AH109 harbors vectors pME5464 (GAL4-AD-VeA$_{1-200}$) and pME5468 (GAL4-BD-VelB^{G240A}).	This study
AH109-VelB^{L331A}-VeA$_{1-200}$	AH109 harbors vectors pME5464 (GAL4-AD-VeA$_{1-200}$) and pME5469 (GAL4-BD-VelB^{L331A}).	This study
AH109-VelB^{G240A,L331A}-VeA$_{1-200}$	AH109 harbors vectors pME5464 (GAL4-AD-VeA$_{1-200}$) and pME5470 (GAL4-BD-VelB^{G240A,L331A}).	This study
AH109-VelB-VelB	AH109 harbors vectors pME5465 (GAL4-AD-VelB) and pME5467 (GAL4-BD-VelB).	This study

AH109-VelB^{G240A}-VelB	AH109 harbors vectors pME5465 (GAL4-AD-VelB) and pME5468 (GAL4-BD-VelB^{G240A}).	This study
AH109-VelB^{L331A}-VelB	AH109 harbors vectors pME5465 (GAL4-AD-VelB) and pME5469 (GAL4-BD-VelB^{L331A}).	This study
AH109-VelB^{G240A,L331A}-VelB	AH109 harbors vectors pME5465 (GAL4-AD-VelB) and pME5470 (GAL4-BD-VelB^{G240A,L331A}).	This study
AH109-VelB-VosA$_{1-190}$	AH109 harbors vectors pME5466 (GAL4-AD-VosA$_{1-190}$) and pME5467 (GAL4-BD-VelB).	This study
AH109-VelB^{G240A}-VosA$_{1-190}$	AH109 harbors vectors pME5466 (GAL4-AD-VosA$_{1-190}$) and pME5468 (GAL4-BD-VelB^{G240A}).	This study
AH109-VelB^{L331A}-VosA$_{1-190}$	AH109 harbors vectors pME5466 (GAL4-AD-VosA$_{1-190}$) and pME5469 (GAL4-BD-VelB^{L331A}).	This study
AH109-VelB^{G240A,L331A}-VosA$_{1-190}$	AH109 harbors vectors pME5466 (GAL4-AD-VosA$_{1-190}$) and pME5470 (GAL4-BD-VelB^{G240A,L331A}).	This study

Materials and Methods

This part provides the detailed information on generating the plasmids and mutants used in this study.

Construction of plasmid pME5332

3'UTR of *velB* was cloned from pME4687 with primers WC22 and WC23 and then integrated in the *Pml*I restriction site of plasmid pME4319 to generate pME5332. pME5332 was used as parental plasmid for the construction of subsequent plasmids.

Construction of plasmid pME5333 and *A. nidulans* mutant of *velBcDNA* (AGB1479)

The *velB* 5' UTR and 5' fragment cloned with primers WC30 and WC151 from template pME4687, *velB* cDNA main part cloned from pME3717 with primers WC84 and WC149 and the remaining *velB* cDNA end: sgfp cloned from pME4687 with primers WC12 and WC150 were integrated into the *Swa*I restriction cutting site of plasmid pME5332 to generate pME5333. The linear *velBcDNA:sgfp: phleoRM* cassette from the digestion of pME5333 by *Pme*I was integrated into AGB1064, resulting in AGB1479.

Construction of plasmid pME5354 and *A. nidulans* mutant of $velB^{L238A}$ (AGB1500)

The *velB* 5' UTR and 5' fragment cloned with primers WC30 and WC94, and the remaining 3' fragment fused with *sgfp* cloned with primers WC12 and WC95 from template pME4687 were integrated into the *Swa*I restriction cutting site of plasmid pME5332 to generate pME5354. The linear $velB^{L238A}$*:sgfp: phleoRM* cassette from the digestion of pME5354 by *Pme*I was integrated into AGB1064, resulting in AGB1500.

Construction of plasmid pME5355 and *A. nidulans* mutant of $velB^{G240A}$ (AGB1501)

The *velB* 5' UTR and 5' fragment cloned with primers WC30 and WC54, and the remaining 3’ fragment fused with *sgfp* cloned with primers WC12 and WC55 from template pME4687 were integrated into the *Swa*I restriction cutting site of plasmid pME5332 to generate pME5355. The linear *velB*^{G240A}*:sgfp: phleoRM* cassette from the digestion of pME5355 by *Pme*I was integrated into AGB1064, resulting in AGB1501.

Construction of plasmid pME5356 and *A. nidulans* mutant of *velB*^{D251A} (AGB1502)

The *velB* 5' UTR and 5' fragment cloned with primers WC30 and WC56, and the remaining 3’ fragment fused with sgfp cloned with primers WC12 and WC57 from template pME4687 were integrated into the SwaI restriction cutting site of plasmid pME5332 to generate pME5356. The linear *velB*^{D251A}*:sgfp: phleoRM* cassette from the digestion of pME5356 by *Pme*I was integrated into AGB1064, resulting in AGB1502.

Construction of plasmid pME5357 and *A. nidulans* mutant of *velB*^{F260A} (AGB1503)

The *velB* 5' UTR and 5' fragment cloned with primers WC30 and WC58, and the remaining 3’ fragment fused with sgfp cloned with primers WC12 and WC59 from template pME4687 were integrated into the SwaI restriction cutting site of plasmid pME5332 to generate pME5357. The linear *velB*^{F260A}*:sgfp: phleoRM* cassette from the digestion of pME5357 by *Pme*I was integrated into AGB1064, resulting in AGB1503.

Construction of plasmid pME5358 and *A. nidulans* mutant of *velB*^{D264A} (AGB1504)

The *velB* 5' UTR and 5' fragment cloned with primers WC30 and WC60, and the remaining 3’ fragment fused with sgfp cloned with primers WC12 and WC61 from template pME4687 were integrated into the *Swa*I restriction cutting site of plasmid pME5332 to generate pME5358. The linear *velB*^{D264A}*:sgfp: phleoRM* cassette from

the digestion of pME5358 by *Pme*I was integrated into AGB1064, resulting in AGB1504.

Construction of plasmid pME5359 and *A. nidulans* mutant of *velB*^{L265A} (AGB1505)

The *velB* 5' UTR and 5' fragment cloned with primers WC30 and WC96, and the remaining 3' fragment fused with *sgfp* cloned with primers WC12 and WC97 from template pME4687 were integrated into the *Swa*I restriction cutting site of plasmid pME5332 to generate pME5359. The linear *velB*^{L265A}*:sgfp: phleoRM* cassette from the digestion of pME5359 by *Pme*I was integrated into AGB1064, resulting in AGB1505.

Construction of plasmid pME5360 and *A. nidulans* mutant of *velB*^{S266A} (AGB1506)

The *velB* 5' UTR and 5' fragment cloned with primers WC30 and WC62, and the remaining 3' fragment fused with *sgfp* cloned with primers WC12 and WC63 from template pME4687 were integrated into the *Swa*I restriction cutting site of plasmid pME5332 to generate pME5360. The linear *velB*^{S266A}*:sgfp: phleoRM* cassette from the digestion of pME5360 by *Pme*I was integrated into AGB1064, resulting in AGB1506.

Construction of plasmid pME5361 and *A. nidulans* mutant of *velB*^{V267A} (AGB1507)

The *velB* 5' UTR and 5' fragment cloned with primers WC30 and WC253, and the remaining 3' fragment fused with *sgfp* cloned with primers WC12 and WC254 from template pME4687 were integrated into the *Swa*I restriction cutting site of plasmid pME5332 to generate pME5361. The linear *velB*^{V267A}*:sgfp: phleoRM* cassette from the digestion of pME5361 by *Pme*I was integrated into AGB1064, resulting in AGB1507.

Construction of plasmid pME5362 and *A. nidulans* mutant of *velB*^{R268A} (AGB1508)

The *velB* 5' UTR and 5' fragment cloned with primers WC30 and WC64, and the remaining 3’ fragment fused with sgfp cloned with primers WC12 and WC65 from template pME4687 were integrated into the SwaI restriction cutting site of plasmid pME5332 to generate pME5362. The linear *velB*^{R268A}*:sgfp: phleoRM* cassette from the digestion of pME5362 by *Pme*I was integrated into AGB1064, resulting in AGB1508.

Construction of plasmid pME5363 and *A. nidulans* mutant of *velB*^{E270A} (AGB1509)

The *velB* 5' UTR and 5' fragment cloned with primers WC30 and WC255, and the remaining 3’ fragment fused with sgfp cloned with primers WC12 and WC256 from template pME4687 were integrated into the SwaI restriction cutting site of plasmid pME5332 to generate pME5363. The linear *velB*^{E270A}*:sgfp: phleoRM* cassette from the digestion of pME5363 by *Pme*I was integrated into AGB1064, resulting in AGB1509.

Construction of plasmid pME5364 and *A. nidulans* mutant of *velB*^{G271A} (AGB1510)

The *velB* 5' UTR and 5' fragment cloned with primers WC30 and WC66, and the remaining 3’ fragment fused with sgfp cloned with primers WC12 and WC67 from template pME4687 were integrated into the *Swa*I restriction cutting site of plasmid pME5332 to generate pME5364. The linear *velB*^{G271A}*:sgfp: phleoRM* cassette from the digestion of pME5364 by *Pme*I was integrated into AGB1064, resulting in AGB1510.

Construction of plasmid pME5365 and *A. nidulans* mutant of *velB*^{F273A} (AGB1511)

The *velB* 5' UTR and 5' fragment cloned with primers WC30 and WC68, and the remaining 3’ fragment fused with *sgfp* cloned with primers WC12 and WC69 from template pME4687 were integrated into the *Swa*I restriction cutting site of plasmid pME5332 to generate pME5365. The linear *velB*^{F273A}*:sgfp: phleoRM* cassette from

the digestion of pME5365 by *Pme*I was integrated into AGB1064, resulting in AGB1511.

Construction of plasmid pME5366 and *A. nidulans* mutant of *velB*^{L275A} (AGB1512)

The *velB* 5' UTR and 5' fragment cloned with primers WC30 and WC70, and the remaining 3' fragment fused with *sgfp* cloned with primers WC12 and WC71 from template pME4687 were integrated into the *Swa*I restriction cutting site of plasmid pME5332 to generate pME5366. The linear *velB*^{L275A}*:sgfp: phleoRM* cassette from the digestion of pME5366 by *Pme*I was integrated into AGB1064, resulting in AGB1512.

Construction of plasmid pME5367 and *A. nidulans* mutant of *velB*^{F277A} (AGB1513)

The *velB* 5' UTR and 5' fragment cloned with primers WC30 and WC72, and the remaining 3' fragment fused with *sgfp* cloned with primers WC12 and WC73 from template pME4687 were integrated into the SwaI restriction cutting site of plasmid pME5332 to generate pME5367. The linear *velB*^{F277A}*:sgfp: phleoRM* cassette from the digestion of pME5367 by *Pme*I was integrated into AGB1064, resulting in AGB1513.

Construction of plasmid pME5368 and *A. nidulans* mutant of *velB*^{F279A} (AGB1514)

The *velB* 5' UTR and 5' fragment cloned with primers WC30 and WC74, and the remaining 3' fragment fused with *sgfp* cloned with primers WC12 and WC75 from template pME4687 were integrated into the *Swa*I restriction cutting site of plasmid pME5332 to generate pME5368. The linear *velB*^{F279A}*:sgfp: phleoRM* cassette from the digestion of pME5368 by *Pme*I was integrated into AGB1064, resulting in AGB1514.

Construction of plasmid pME5369 and *A. nidulans* mutant of *velB*^{S311A} (AGB1515)

The *velB* 5' UTR and 5' fragment cloned with primers WC30 and WC76, and the remaining 3’ fragment fused with *sgfp* cloned with primers WC12 and WC77 from template pME4687 were integrated into the *Swa*I restriction cutting site of plasmid pME5332 to generate pME5369. The linear *velB*^{S311A}*:sgfp: phleoRM* cassette from the digestion of pME5369 by *Pme*I was integrated into AGB1064, resulting in AGB1515.

Construction of plasmid pME5370 and *A. nidulans* mutant of *velB*^{F314A} (AGB1516)

The *velB* 5' UTR and 5' fragment cloned with primers WC30 and WC78, and the remaining 3’ fragment fused with *sgfp* cloned with primers WC12 and WC79 from template pME4687 were integrated into the *Swa*I restriction cutting site of plasmid pME5332 to generate pME5370. The linear *velB*^{F314A}*:sgfp: phleoRM* cassette from the digestion of pME5370 by *Pme*I was integrated into AGB1064, resulting in AGB1516.

Construction of plasmid pME5371 and *A. nidulans* mutant of *velB*^{V316A} (AGB1517)

The *velB* 5' UTR and 5' fragment cloned with primers WC30 and WC80, and the remaining 3’ fragment fused with *sgfp* cloned with primers WC12 and WC81 from template pME4687 were integrated into the *Swa*I restriction cutting site of plasmid pME5332 to generate pME5371. The linear *velB*^{V316A}*:sgfp: phleoRM* cassette from the digestion of pME5371 by *Pme*I was integrated into AGB1064, resulting in AGB1517.

Construction of plasmid pME5372 and *A. nidulans* mutant of *velB*^{K320A} (AGB1518)

The *velB* 5' UTR and 5' fragment cloned with primers WC30 and WC82, and the remaining 3’ fragment fused with sgfp cloned with primers WC12 and WC83 from template pME4687 were integrated into the *Swa*I restriction cutting site of plasmid pME5332 to generate pME5372. The linear *velB*^{K320A}*:sgfp: phleoRM* cassette from

the digestion of pME5372 by *Pme*I was integrated into AGB1064, resulting in AGB1518.

Construction of plasmid pME5373 and *A. nidulans* mutant of *velB*^{F322A} (AGB1519)

The *velB* 5' UTR and 5' fragment cloned with primers WC30 and WC84, and the remaining 3' fragment fused with *sgfp* cloned with primers WC12 and WC85 from template pME4687 were integrated into the *Swa*I restriction cutting site of plasmid pME5332 to generate pME5373. The linear *velB*^{F322A}*:sgfp: phleoRM* cassette from the digestion of pME5373 by *Pme*I was integrated into AGB1064, resulting in AGB1519.

Construction of plasmid pME5374 and *A. nidulans* mutant of *velB*^{P323A} (AGB1520)

The *velB* 5' UTR and 5' fragment cloned with primers WC30 and WC86, and the remaining 3' fragment fused with *sgfp* cloned with primers WC12 and WC87 from template pME4687 were integrated into the SwaI restriction cutting site of plasmid pME5332 to generate pME5374. The linear *velB*^{P323A}*:sgfp: phleoRM* cassette from the digestion of pME5374 by *Pme*I was integrated into AGB1064, resulting in AGB1520.

Construction of plasmid pME5375 and *A. nidulans* mutant of *velB*^{G324A} (AGB1521)

The *velB* 5' UTR and 5' fragment cloned with primers WC30 and WC88, and the remaining 3' fragment fused with sgfp cloned with primers WC12 and WC89 from template pME4687 were integrated into the *Swa*I restriction cutting site of plasmid pME5332 to generate pME5375. The linear *velB*^{G324A}*:sgfp: phleoRM* cassette from the digestion of pME5375 by *Pme*I was integrated into AGB1064, resulting in AGB1521.

Construction of plasmid pME5376 and *A. nidulans* mutant of *velB*^{S328A} (AGB1522)

The *velB* 5' UTR and 5' fragment cloned with primers WC30 and WC90, and the remaining 3' fragment fused with sgfp cloned with primers WC12 and WC91 from template pME4687 were integrated into the SwaI restriction cutting site of plasmid pME5332 to generate pME5376. The linear $velB^{S328A}$*:sgfp: phleoRM* cassette from the digestion of pME5376 by *Pme*I was integrated into AGB1064, resulting in AGB1522.

Construction of plasmid pME5377 and *A. nidulans* mutant of $velB^{T329A}$ (AGB1523)

The *velB* 5' UTR and 5' fragment cloned with primers WC30 and WC98, and the remaining 3' fragment fused with *sgfp* cloned with primers WC12 and WC99 from template pME4687 were integrated into the *Swa*I restriction cutting site of plasmid pME5332 to generate pME5377. The linear $velB^{T329A}$*:sgfp: phleoRM* cassette from the digestion of pME5377 by *Pme*I was integrated into AGB1064, resulting in AGB1523.

Construction of plasmid pME5378 and *A. nidulans* mutant of $velB^{L331A}$ (AGB1524)

The *velB* 5' UTR and 5' fragment cloned with primers WC30 and WC92, and the remaining 3' fragment fused with *sgfp* cloned with primers WC12 and WC93 from template pME4687 were integrated into the *Swa*I restriction cutting site of plasmid pME5332 to generate pME5378. The linear $velB^{L331A}$:sgfp: phleoRM cassette from the digestion of pME5378 by *Pme*I was integrated into AGB1064, resulting in AGB1524.

Construction of plasmid pME5379 and *A. nidulans* mutant of $velB^{Q338A}$ (AGB1525)

The *velB* 5' UTR and 5' fragment cloned with primers WC30 and WC100, and the remaining 3' fragment fused with *sgfp* cloned with primers WC12 and WC101 from template pME4687 were integrated into the *Swa*I restriction cutting site of plasmid pME5332 to generate pME5379. The linear $velB^{Q338A}$*:sgfp: phleoRM* cassette from

the digestion of pME5379 by *Pme*I was integrated into AGB1064, resulting in AGB1525.

Construction of plasmid pME5380 and *A. nidulans* mutant of *velB*^{G339A} (AGB1526)

The *velB* 5' UTR and 5' fragment cloned with primers WC30 and WC102, and the remaining 3' fragment fused with *sgfp* cloned with primers WC12 and WC103 from template pME4687 were integrated into the *Swa*I restriction cutting site of plasmid pME5332 to generate pME5380. The linear velB^{G339A}:sgfp: phleoRM cassette from the digestion of pME5380 by *Pme*I was integrated into AGB1064, resulting in AGB1526.

Construction of plasmid pME5381 and *A. nidulans* mutant of *velB*^{K341A} (AGB1527)

The *velB* 5' UTR and 5' fragment cloned with primers WC30 and WC104, and the remaining 3' fragment fused with *sgfp* cloned with primers WC12 and WC105 from template pME4687 were integrated into the *Swa*I restriction cutting site of plasmid pME5332 to generate pME5381. The linear *velB*^{K341A}*:sgfp: phleoRM* cassette from the digestion of pME5381 by *Pme*I was integrated into AGB1064, resulting in AGB1527.

Construction of plasmid pME5382 and *A. nidulans* mutant of *velB*^{I344A} (AGB1528)

The *velB* 5' UTR and 5' fragment cloned with primers WC30 and WC106, and the remaining 3' fragment fused with *sgfp* cloned with primers WC12 and WC107 from template pME4687 were integrated into the *Swa*I restriction cutting site of plasmid pME5332 to generate pME5382. The linear *velB*^{I344A}*:sgfp: phleoRM* cassette from the digestion of pME5382 by *Pme*I was integrated into AGB1064, resulting in AGB1528.

Construction of plasmid pME5383 and *A. nidulans* mutant of *velB*^{R345A} (AGB1529)

The *velB* 5' UTR and 5' fragment cloned with primers WC30 and WC108, and the remaining 3' fragment fused with *sgfp* cloned with primers WC12 and WC109 from template pME4687 were integrated into the *Swa*I restriction cutting site of plasmid pME5332 to generate pME5383. The linear *velB*^{R345A}*:sgfp: phleoRM* cassette from the digestion of pME5383 by *Pme*I was integrated into AGB1064, resulting in AGB1529.

Construction of plasmid pME5393 and *A. nidulans* mutant of *velB*$^{\Delta D288}$ (AGB1540)

The *velB* 5' UTR and 5' fragment cloned with primers WC30 and WC202, and the remaining 3' fragment fused with *sgfp* cloned with primers WC12 and WC203 from template pME5333 were integrated into the *Swa*I restriction cutting site of plasmid pME5332 to generate pME5393. The linear *velB*$^{\Delta D288}$*:sgfp: phleoRM* cassette from the digestion of pME5393 by *Pme*I was integrated into AGB1064, resulting in AGB1540.

Construction of plasmid pME5394 and *A. nidulans* mutant of *velB*$^{\Delta L289}$ (AGB1541)

The *velB* 5' UTR and 5' fragment cloned with primers WC30 and WC165, and the remaining 3' fragment fused with *sgfp* cloned with primers WC12 and WC166 from template pME5333 were integrated into the *Swa*I restriction cutting site of plasmid pME5332 to generate pME5394. The linear *velB*$^{\Delta L289}$*:sgfp: phleoRM* cassette from the digestion of pME5394 by *Pme*I was integrated into AGB1064, resulting in AGB1541.

Construction of plasmid pME5395 and *A. nidulans* mutant of *velB*$^{\Delta LP289\text{-}290}$ (AGB1542)

The *velB* 5' UTR and 5' fragment cloned with primers WC30 and WC167, and the remaining 3' fragment fused with *sgfp* cloned with primers WC12 and WC168 from template pME5333 were integrated into the *Swa*I restriction cutting site of plasmid pME5332 to generate pME5395. The linear *velB*$^{\Delta LP289\text{-}290}$*:sgfp: phleoRM* cassette

from the digestion of pME5395 by *Pme*I was integrated into AGB1064, resulting in AGB1542.

Construction of plasmid pME5396 and *A. nidulans* mutant of *velB*$^{\Delta LPQ289\text{-}291}$ (AGB1543)

The *velB* 5' UTR and 5' fragment cloned with primers WC30 and WC169, and the remaining 3' fragment fused with *sgfp* cloned with primers WC12 and WC170 from template pME5333 were integrated into the *Swa*I restriction cutting site of plasmid pME5332 to generate pME5396. The linear *velB*$^{\Delta LPQ289\text{-}291}$*:sgfp: phleoRM* cassette from the digestion of pME5396 by *Pme*I was integrated into AGB1064, resulting in AGB1543.

Construction of plasmid pME5397 and *A. nidulans* mutant of *velB*$^{\Delta LPQS289\text{-}292}$ (AGB1544)

The *velB* 5' UTR and 5' fragment cloned with primers WC30 and WC196, and the remaining 3' fragment fused with *sgfp* cloned with primers WC12 and WC197 from template pME5333 were integrated into the *Swa*I restriction cutting site of plasmid pME5332 to generate pME5397. The linear *velB*$^{\Delta LPQS289\text{-}292}$*:sgfp: phleoRM* cassette from the digestion of pME5397 by *Pme*I was integrated into AGB1064, resulting in AGB1544.

Construction of plasmid pME5398 and *A. nidulans* mutant of *velB*$^{\Delta LPQSD289\text{-}293}$ (AGB1545)

The *velB* 5' UTR and 5' fragment cloned with primers WC30 and WC198, and the remaining 3' fragment fused with *sgfp* cloned with primers WC12 and WC199 from template pME5333 were integrated into the *Swa*I restriction cutting site of plasmid pME5332 to generate pME5398. The linear *velB*$^{\Delta LPQSD289\text{-}293}$*:sgfp: phleoRM* cassette from the digestion of pME5398 by *Pme*I was integrated into AGB1064, resulting in AGB1545.

Construction of plasmid pME5399 and *A. nidulans* mutant of *velB*$^{\Delta LPQSDI289\text{-}294}$ (AGB1546)

The *velB* 5' UTR and 5' fragment cloned with primers WC30 and WC200, and the remaining 3' fragment fused with *sgfp* cloned with primers WC12 and WC201 from template pME5333 were integrated into the *Swa*I restriction cutting site of plasmid pME5332 to generate pME5399. The linear *velB*$^{\Delta LPQSDI289\text{-}294}$*:sgfp: phleoRM* cassette from the digestion of pME5399 by *Pme*I was integrated into AGB1064, resulting in AGB1546.

Construction of plasmid pME5400 and *A. nidulans* mutant of *velB*$^{\Delta LPQSDIA289\text{-}295}$ (AGB1547)

The *velB* 5' UTR and 5' fragment cloned with primers WC30 and WC214, and the remaining 3' fragment fused with *sgfp* cloned with primers WC12 and WC215 from template pME5333 were integrated into the *Swa*I restriction cutting site of plasmid pME5332 to generate pME5400. The linear *velB*$^{\Delta LPQSDIA289\text{-}295}$*:sgfp: phleoRM* cassette from the digestion of pME5400 by *Pme*I was integrated into AGB1064, resulting in AGB1547.

Construction of plasmid pME5401 and *A. nidulans* mutant of *velB*$^{\Delta LPQSDIAE289\text{-}296}$ (AGB1548)

The *velB* 5' UTR and 5' fragment cloned with primers WC30 and WC216, and the remaining 3' fragment fused with *sgfp* cloned with primers WC12 and WC217 from template pME5333 were integrated into the *Swa*I restriction cutting site of plasmid pME5332 to generate pME5401. The linear *velB*$^{\Delta LPQSDIAE289\text{-}296}$*:sgfp: phleoRM* cassette from the digestion of pME5401 by *Pme*I was integrated into AGB1064, resulting in AGB1548.

Construction of plasmid pME5402 and *A. nidulans* mutant of *velB*$^{\Delta LPQSDIAEV289\text{-}297}$ (AGB1549)

The *velB* 5' UTR and 5' fragment cloned with primers WC30 and WC218, and the remaining 3' fragment fused with *sgfp* cloned with primers WC12 and WC219 from template pME5333 were integrated into the *Swa*I restriction cutting site of plasmid pME5332 to generate pME5402. The linear *velB*$^{\Delta LPQSDIAEV289\text{-}297}$*:sgfp: phleoRM*

cassette from the digestion of pME5402 by *Pme*I was integrated into AGB1064, resulting in AGB1549.

Construction of plasmid pME5403 and *A. nidulans* mutant of *velB*$^{\Delta LPQSDIAEVI289\text{-}298}$ (AGB1550)

The *velB* 5' UTR and 5' fragment cloned with primers WC30 and WC239, and the remaining 3' fragment fused with *sgfp* cloned with primers WC12 and WC240 from template pME5333 were integrated into the *Swa*I restriction cutting site of plasmid pME5332 to generate pME5403. The linear *velB*$^{\Delta LPQSDIAEVI289\text{-}298}$*:sgfp: phleoRM* cassette from the digestion of pME5403 by *Pme*I was integrated into AGB1064, resulting in AGB1550.

Construction of plasmid pME5404 and *A. nidulans* mutant of *velB*$^{\Delta LPQSDIAEVIN289\text{-}299}$ (AGB1551)

The *velB* 5' UTR and 5' fragment cloned with primers WC30 and WC241, and the remaining 3' fragment fused with *sgfp* cloned with primers WC12 and WC242 from template pME5333 were integrated into the *Swa*I restriction cutting site of plasmid pME5332 to generate pME5404. The linear *velB*$^{\Delta LPQSDIAEVIN289\text{-}299}$*:sgfp: phleoRM* cassette from the digestion of pME5404 by *Pme*I was integrated into AGB1064, resulting in AGB1551.

Construction of plasmid pME5405 and *A. nidulans* mutant of *velB*$^{\Delta LPQSDIAEVINK289\text{-}300}$ (AGB1552)

The *velB* 5' UTR and 5' fragment cloned with primers WC30 and WC243, and the remaining 3' fragment fused with *sgfp* cloned with primers WC12 and WC244 from template pME5333 were integrated into the *Swa*I restriction cutting site of plasmid pME5332 to generate pME5405. The linear *velB*$^{\Delta LPQSDIAEVINK289\text{-}300}$*:sgfp: phleoRM* cassette from the digestion of pME5405 by *Pme*I was integrated into AGB1064, resulting in AGB1552.

Construction of plasmid pME5406 and *A. nidulans* mutant of *velB*$^{\Delta LPQSDIAEVINKG289\text{-}301}$ (AGB1553)

The *velB* 5' UTR and 5' fragment cloned with primers WC30 and WC245, and the remaining 3’ fragment fused with *sgfp* cloned with primers WC12 and WC246 from template pME5333 were integrated into the *Swa*I restriction cutting site of plasmid pME5332 to generate pME5406. The linear *velB*$^{\Delta LPQSDIAEVINKG289\text{-}301}$*:sgfp: phleoRM* cassette from the digestion of pME5406 by *Pme*I was integrated into AGB1064, resulting in AGB1553.

Construction of plasmid pME5407 and *A. nidulans* mutant of *velB*$^{\Delta LPQSDIAEVINKGT289\text{-}302}$ (AGB1554)

The *velB* 5' UTR and 5' fragment cloned with primers WC30 and WC247, and the remaining 3’ fragment fused with *sgfp* cloned with primers WC12 and WC248 from template pME5333 were integrated into the *Swa*I restriction cutting site of plasmid pME5332 to generate pME5407. The linear *velB*$^{\Delta LPQSDIAEVINKGT289\text{-}302}$*:sgfp: phleoRM* cassette from the digestion of pME5407 by *Pme*I was integrated into AGB1064, resulting in AGB1554.

Construction of plasmid pME5408 and *A. nidulans* mutant of *velB*$^{\Delta LPQSDIAEVINKGTA289\text{-}303}$ (AGB1555)

The *velB* 5' UTR and 5' fragment cloned with primers WC30 and WC249, and the remaining 3’ fragment fused with *sgfp* cloned with primers WC12 and WC250 from template pME5333 were integrated into the *Swa*I restriction cutting site of plasmid pME5332 to generate pME5408. The linear *velB*$^{\Delta LPQSDIAEVINKGTA289\text{-}303}$*:sgfp: phleoRM* cassette from the digestion of pME5408 by *Pme*I was integrated into AGB1064, resulting in AGB1555.

Construction of plasmid pME5409 and *A. nidulans* mutant of *velB*$^{\Delta S287}$ (AGB1556)

The *velB* 5' UTR and 5' fragment cloned with primers WC30 and WC204, and the remaining 3’ fragment fused with *sgfp* cloned with primers WC12 and WC205 from template pME5333 were integrated into the *Swa*I restriction cutting site of plasmid pME5332 to generate pME5409. The linear *velB*$^{\Delta S287}$*:sgfp: phleoRM* cassette from

the digestion of pME5409 by *Pme*I was integrated into AGB1064, resulting in AGB1556.

Construction of plasmid pME5410 and *A. nidulans* mutant of *velB*$^{\Delta VS286\text{-}287}$ (AGB1557)

The *velB* 5' UTR and 5' fragment cloned with primers WC30 and WC206, and the remaining 3' fragment fused with *sgfp* cloned with primers WC12 and WC207 from template pME5333 were integrated into the *Swa*I restriction cutting site of plasmid pME5332 to generate pME5410. The linear *velB*$^{\Delta VS286\text{-}287}$*:sgfp: phleoRM* cassette from the digestion of pME5410 by PmeI was integrated into AGB1064, resulting in AGB1557.

Construction of plasmid pME5411 and *A. nidulans* mutant of *velB*$^{\Delta SVS285\text{-}287}$ (AGB1558)

The *velB* 5' UTR and 5' fragment cloned with primers WC30 and WC220, and the remaining 3' fragment fused with *sgfp* cloned with primers WC12 and WC221 from template pME5333 were integrated into the *Swa*I restriction cutting site of plasmid pME5332 to generate pME5411. The linear *velB*$^{\Delta SVS285\text{-}287}$*:sgfp: phleoRM* cassette from the digestion of pME5411 by *Pme*I was integrated into AGB1064, resulting in AGB1558.

Construction of plasmid pME5412 and *A. nidulans* mutant of *velB*$^{\Delta KSVS284\text{-}287}$ (AGB1559)

The *velB* 5' UTR and 5' fragment cloned with primers WC30 and WC222, and the remaining 3' fragment fused with *sgfp* cloned with primers WC12 and WC223 from template pME5333 were integrated into the *Swa*I restriction cutting site of plasmid pME5332 to generate pME5412. The linear *velB*$^{\Delta KSVS284\text{-}287}$*:sgfp: phleoRM* cassette from the digestion of pME5412 by *Pme*I was integrated into AGB1064, resulting in AGB1559.

Construction of plasmid pME5413 and *A. nidulans* mutant of *velB*$^{\Delta GKSVS283\text{-}287}$ (AGB1560)

The *velB* 5' UTR and 5' fragment cloned with primers WC30 and WC224, and the remaining 3’ fragment fused with *sgfp* cloned with primers WC12 and WC225 from template pME5333 were integrated into the *Swa*I restriction cutting site of plasmid pME5332 to generate pME5413. The linear *velB*$^{\Delta GKSVS283\text{-}287}$*:sgfp: phleoRM* cassette from the digestion of pME5413 by *Pme*I was integrated into AGB1064, resulting in AGB1560.

Construction of plasmid pME5414 and *A. nidulans* mutant of *velB*$^{\Delta VGKSVS282\text{-}287}$ (AGB1561)

The *velB* 5' UTR and 5' fragment cloned with primers WC30 and WC233, and the remaining 3’ fragment fused with *sgfp* cloned with primers WC12 and WC234 from template pME5333 were integrated into the *Swa*I restriction cutting site of plasmid pME5332 to generate pME5414. The linear *velB*$^{\Delta VGKSVS282\text{-}287}$*:sgfp: phleoRM* cassette from the digestion of pME5414 by *Pme*I was integrated into AGB1064, resulting in AGB1561.

Construction of plasmids pME5464-pME5470

The *veA*$_{1\text{-}200}$ cDNA cloned with primers WC286 and WC287 from template pME3189 was integrated into the SmaI restriction cutting site of plasmid pGADT7 to generate pME5464.

The *velB* cDNA cloned with primers WC282 and WC283 from template pME3189 was integrated into the SmaI restriction cutting site of plasmid pGADT7 to generate pME5465.

The *vosA*$_{1\text{-}190}$ cDNA cloned with primers WC284 and WC285 from template pME3715 was integrated into the SmaI restriction cutting site of plasmid pGADT7 to generate pME5466. The *velB* cDNA cloned with primers WC278 and WC279 from template pME3189 was integrated into the SmaI restriction cutting site of plasmid pGBKT7 to generate pME5467.

A two-step cloning strategy was used for production of *velB*^{G240A}, *velB*^{L331A} and *velB*$^{G240A,\ L331A}$ cDNAs. In the first step, the *velB* 5' UTR and 5' fragment cloned

with primers WC30 and WC54, and the remaining 3' part fused with sgfp cloned with primers WC12 and WC55 from template pME3189 were integrated into the SwaI restriction cutting site of plasmid pME5332. In the second step, this new plasmid was used to clone *velB*^{G240A} cDNA with primers WC278 and WC279 and the recycled PCR product was integrated into the SmaI restriction cutting site of plasmid pGBKT7 to generate pME5468. The *velB*^{L331A} cDNA was clone with the same process by mutated primers WC92 and WC93, and integrated into the SmaI restriction cutting site of plasmid pGBKT7 to generate pME5469. The *velB*$^{G240A, L331A}$ cDNA was clone from the above plasmid containing *velB*^{L331A} cDNA with primers WC54 and WC55, and integrated into the SmaI restriction cutting site of plasmid pGBKT7 to generate pME5470.

Construction of plasmid pME5471 and *A. nidulans* mutant of *velB*^{G240A,L331A} (AGB1637)

The *velB* 5' UTR and 5' fragment cloned with primers WC30 and WC54, and the remaining 3' part fused with *sgfp* cloned with primers WC12 and WC55 from template pME5378 were integrated into the *Swa*I restriction cutting site of plasmid pME5332 by seamless cloning to generate pME5471. The linear *velB*$^{G240A, L331A}$*:sgfp: phleoRM* cassette from the digestion of pME5471 by *Pme*I was integrated into AGB1064, resulting in AGB1637.

Construction of plasmid pME5472 and *A. nidulans* mutant of *veA*^{G83A} (AGB1638)

The two-step cloning strategy was used for production of pME5472. In the first step, 3'UTR of *veA* was cloned from pME4714 with primers WC20 and WC21 and integrated in the *Pml*I restriction cutting site of plasmid pME4319. In the second step, the *veA* 5' UTR and 5' fragment cloned with primers WC30 and WC289, and the remaining 3' part fused with *sgfp* cloned with primers WC12 and WC290 from the template pME4714 were integrated into the SwaI restriction cutting site of the plasmid constructed in the first step to generate pME5472. The linear *veA*^{G83A}*:sgfp: phleoRM* cassette from the digestion of pME5472 by *Pme*I was integrated into AGB1066, resulting in AGB1638.

Construction of plasmid pME5473 and *A. nidulans* mutant of *veA*^{I180A} (AGB1639)

pME5473 was generated by the two-step cloning strategy. In the first step, 3'UTR of *veA* was cloned from pME4714 with primers WC20 and WC21 and integrated in the *Pml*I restriction cutting site of plasmid pME4319. In the second step, the *veA* 5' UTR and 5' fragment cloned with primers WC30 and WC291, and the remaining 3' part fused with sgfp cloned with primers WC12 and WC292 from the template pME4714 were integrated into the SwaI restriction cutting site of the plasmid constructed in the first step to generate pME5473. The linear *veA*^{I180A}*:sgfp: phleoRM* cassette from the digestion of pME5473 by *Pme*I was integrated into AGB1066, resulting in AGB1639.

Construction of plasmid pME5474 and *A. nidulans* mutant of *veA*^{G83A,I180A} (AGB1640)

pME5474 was generated by the two-step cloning strategy. In the first step, 3'UTR of *veA* was cloned from pME4714 with primers WC20 and WC21 and integrated in the *Pml*I restriction cutting site of plasmid pME4319. In the second step, the *veA* 5' UTR and 5' fragment cloned with primers WC30 and WC289, and the remaining 3' part fused with *sgfp* cloned with primers WC12 and WC290 from the template pME5473 were integrated into the *Swa*I restriction cutting site of the plasmid constructed in the first step to generate pME5474. The linear *veA*^{G83A,I180A}*:sgfp: phleoRM* cassette from the digestion of pME5474 by *Pme*I was integrated into AGB1066, resulting in AGB1640.

Construction of plasmid pME5475 and *A. nidulans* mutant of *vosA*^{G96A} (AGB1641)

pME5475 was generated by the two-step cloning strategy. In the first step, the 3'UTR of *A. nidulans vosA* cloned with primers WC5 and WC6 from the template pME4749 was integrated in the PmlI restriction cutting site of plasmid pME4319. In the second step, the *vosA* 5' UTR and 5' fragment cloned with primers WC1 and WC293, and the remaining 3' part fused with *sgfp* cloned with primers WC12 and

WC294 from the template pME4749 were integrated into the *Swa*I restriction cutting site of the plasmid constructed in the first step to generate pME5475. The linear *vosA*^{G96A}*:sgfp: phleoRM* cassette from the digestion of pME5475 by *Pme*I was integrated into AGB1057, resulting in AGB1641.

Construction of plasmid pME5476 and *A. nidulans* mutant of *vosA*^{L171A} (AGB1642)

pME5476 was generated by the two-step cloning strategy. In the first step, the 3'UTR of *A. nidulans vosA* cloned with primers WC5 and WC6 from the template pME4749 was integrated in the *Pml*I restriction cutting site of plasmid pME4319. In the second step, the *vosA* 5' UTR and 5' fragment cloned with primers WC1 and WC295, and the remaining 3' part fused with *sgfp* cloned with primers WC12 and WC296 from the template pME4749 were integrated into the *Swa*I restriction cutting site of the plasmid constructed in the first step to generate pME5476. The linear *vosA*^{L171A}*:sgfp: phleoRM* cassette from the digestion of pME5476 by PmeI was integrated into AGB1057, resulting in AGB1642.

Construction of plasmid pME5477 and *A. nidulans* mutant of *vosA*^{G96A,L171A} (AGB1643)

pME5477 was generated by the two-step cloning strategy. In the first step, the 3'UTR of *A. nidulans vosA* cloned with primers WC5 and WC6 from the template pME4749 was integrated in the *Pml*I restriction cutting site of plasmid pME4319. In the second step, the *vosA* 5' UTR and 5' fragment cloned with primers WC1 and WC293, and the remaining 3' part fused with *sgfp* cloned with primers WC12 and WC294 from the template pME5476 were integrated into the *Swa*I restriction cutting site of the plasmid constructed in the first step to generate pME5477. The linear *vosA*^{G96A,L171A}*:sgfp: phleoRM* cassette from the digestion of pME5477 by *Pme*I was integrated into AGB1057, resulting in AGB1643.

References

1. Bayram O, Bayram OS, Ahmed YL, Maruyama J, Valerius O, Rizzoli SO, Ficner R, Irniger S, Braus GH. 2012. The *Aspergillus nidulans* mapk module anste11-ste50-ste7-fus3 controls development and secondary metabolism. PLoS Genet 8:e1002816. https://doi:10.1371/journal.pgen.1002816
2. Thieme KG, Gerke J, Sasse C, Valerius O, Thieme S, Karimi R, Heinrich AK, Finkernagel F, Smith K, Bode HB, Freitag M, Ram AFJ, Braus GH. 2018. Velvet domain protein VosA represses the zinc cluster transcription factor SclB regulatory network for *Aspergillus nidulans* asexual development, oxidative stress response and secondary metabolism. PLoS Genet 14:e1007511. https://doi:10.1371/journal.pgen.1007511
3. Meister C, Thieme KG, Thieme S, Kohler AM, Schmitt K, Valerius O, Braus GH. 2019. COP9 signalosome interaction with UspA/Usp15 deubiquitinase controls VeA-mediated fungal multicellular development. Biomolecules 9:238. https://doi:10.3390/biom9060238
4. Thieme S. 2018. Insertion of an intrinsically disordered domain in VelB supports selective heterodimer formation of fungal velvet domain regulatory proteins in *Aspergillus nidulans*. PhD thesis. Georg-August University Göttingen, Göttingen, Germany.
5. Bayram O, Krappmann S, Ni M, Bok JW, Helmstaedt K, Valerius O, Braus-Stromeyer S, Kwon NJ, Keller NP, Yu JH, Braus GH. 2008. VelB/VeA/LaeA complex coordinates light signal with fungal development and secondary metabolism. Science 320:1504-1506. https://doi:10.1126/science.1155888
6. Sarikaya Bayram O, Bayram O, Valerius O, Park HS, Irniger S, Gerke J, Ni M, Han KH, Yu JH, Braus GH. 2010. LaeA control of velvet family regulatory proteins for light-dependent development and fungal cell-type specificity. PLoS Genet 6:e1001226. https://doi:10.1371/journal.pgen.1001226
7. Liu L, Sasse C, Dirnberger B, Valerius O, Fekete-Szücs E, Harting R, Nordzieke DE, Pöggeler S, Karlovsky P, Gerke J, Braus GH. 2021. Secondary metabolites of Hülle cells mediate protection of fungal reproductive and overwintering structures against fungivorous animals.

eLife 10:e68058. https://doi:10.7554/eLife.68058
